# Salvage therapies for biochemical recurrence after definitive local treatment: a systematic review, meta-analysis, and network meta-analysis

**DOI:** 10.1038/s41391-024-00890-4

**Published:** 2024-09-13

**Authors:** Akihiro Matsukawa, Takafumi Yanagisawa, Tamas Fazekas, Marcin Miszczyk, Ichiro Tsuboi, Mehdi Kardoust Parizi, Ekaterina Laukhtina, Jakob Klemm, Stefano Mancon, Keiichiro Mori, Shoji Kimura, Jun Miki, Juan Gomez Rivas, Timo F. W. Soeterik, Thomas Zilli, Derya Tilki, Steven Joniau, Takahiro Kimura, Shahrokh F. Shariat, Pawel Rajwa

**Affiliations:** 1 Department of Urology, Comprehensive Cancer Center, Medical University of Vienna, Vienna, Austria; 2 Department of Urology, The Jikei University School of Medicine, Tokyo, Japan; 3 Department of Urology, Semmelweis University, Budapest, Hungary; 4 Centre for Translational Medicine, Semmelweis University, Budapest, Hungary; 5 Collegium Medicum – Faculty of Medicine, WSB University, Dąbrowa Górnicza, Poland; 6 Department of Urology, Faculty of Medicine, Shimane University, Shimane, Japan; 7 Department of Urology, Shariati Hospital, Tehran University of Medical Science, Tehran, Iran; 8 Institute for Urology and Reproductive Health, Sechenov University, Moscow, Russia; 9 Department of Urology, University Medical Center Hamburg-Eppendorf, Hamburg, Germany; 10 Department of Biomedical Sciences, Humanitas University, Pieve Emanuele, Italy; 11 Department of Urology, Clinico San Carlos Hospital, Madrid, Spain; 12 Department of Urology, St. Antonius Hospital, Utrecht, The Netherlands; 13 Department of Radiation Oncology, University Medical Center Utrecht, Utrecht, The Netherlands; 14 Department of Radiation Oncology, Oncology Institute of Southern Switzerland, Ente Ospedaliero Cantonale, Bellinzona, Switzerland; 15 Faculty of Medicine, University of Geneva, Geneva, Switzerland; 16 Martini-Klinik Prostate Cancer Center, University Hospital Hamburg-Eppendorf, Hamburg, Germany; 17 Department of Urology, Koc University Hospital, Istanbul, Turkey; 18 Department of Urology, University Hospitals Leuven, Leuven, Belgium; 19 Department of Development and regeneration, KU Leuven, Leuven, Belgium; 20 Department of Urology, University of Texas Southwestern Medical Center, Dallas, TX USA; 21 Department of Urology, Weill Cornell Medical College, New York, NY USA; 22 Department of Urology, Second Faculty of Medicine, Charles University, Prague, Czechia; 23 Division of Urology, Department of Special Surgery, The University of Jordan, Amman, Jordan; 24 Karl Landsteiner Institute of Urology and Andrology, Vienna, Austria; 25 Research Center for Evidence Medicine, Urology Department, Tabriz University of Medical Sciences, Tabriz, Iran; 26 Department of Urology, Medical University of Silesia, Zabrze, Poland

**Keywords:** Prostate cancer, Prostate cancer

## Abstract

**Purpose:**

Recent advancements in the management of biochemical recurrence (BCR) following local treatment for prostate cancer (PCa), including the use of androgen receptor signaling inhibitors (ARSIs), have broadened the spectrum of therapeutic options. We aimed to compare salvage therapies in patients with BCR after definitive local treatment for clinically non-metastatic PCa with curative intent.

**Methods:**

In October 2023, we queried PubMed, Scopus, and Web of Science databases to identify randomized controlled trials (RCTs) and prospective studies reporting data on the efficacy of salvage therapies in PCa patients with BCR after radical prostatectomy (RP) or radiation therapy (RT). The primary endpoint was metastatic-free survival (MFS), and secondary endpoints included progression-free survival (PFS) and overall survival (OS).

**Results:**

We included 19 studies (*n* = 9117); six trials analyzed RT-based strategies following RP, ten trials analyzed hormone-based strategies following RP ± RT or RT alone, and three trials analyzed other agents. In a pairwise meta-analysis, adding hormone therapy to salvage RT significantly improved MFS (HR: 0.69, 95% CI: 0.57–0.84, *p* < 0.001) compared to RT alone. Based on treatment ranking analysis, among RT-based strategies, the addition of elective nodal RT and androgen deprivation therapy (ADT) was found to be the most effective in terms of MFS. On the other hand, among hormone-based strategies, enzalutamide + ADT showed the greatest benefit for both MFS and OS.

**Conclusions:**

The combination of prostate bed RT, elective pelvic irradiation, and ADT is the preferred treatment for eligible patients with post-RP BCR based on our analysis. In remaining patients, or in case of post-RT recurrence, especially for those with high-risk BCR, the combination of ADT and ARSI should be considered.

## Introduction

While local treatments such as radical prostatectomy (RP) or radiation therapy (RT) are effective in early-stage prostate cancer (PCa), 20–40% of patients experience biochemical recurrence (BCR) [1]. Approximately one-third of patients with BCR already have detectable metastasis on conventional imaging, which is still likely underestimated considering the results of studies analyzing prostate-specific membrane antigen positron emission tomography (PSMA-PET) in the BCR setting [2, 3]. Although the majority of patients with low-risk BCR are unlikely to develop metastases [4], high-risk BCR is associated with mortality [5–8], highlighting the compelling need to improve BCR management.

Current guidelines recommend RT with or without androgen deprivation therapy (ADT) for patients who experience BCR following RP [9–11]. In addition, ADT is recommended as an option for those experiencing BCR after receiving adjuvant, salvage, or definitive RT. The implementation of androgen receptor signaling inhibitors (ARSIs) and other agents (e.g., docetaxel [DOC]) as an effective treatment for advanced PCa, may also potentially broaden therapeutic choices in BCR [12]. However, despite emerging data, there is a lack of comprehensive synthesis in the literature to guide clinical decision-making for BCR treatment. Therefore, we conducted this systematic review, meta-analysis, and network meta-analysis (NMA) to identify the most effective salvage therapy strategy for patients with BCR following definitive local therapy.

## Methods

Our study protocol is registered with the International Prospective Register of Systemic Reviews database (PROSPERO: CRD42023481828). This meta-analysis adheres to the guidelines of the Preferred Reporting Items for Systematic Reviews and Meta-Analyses (PRISMA) statement and AMSTAR2 checklist [13, 14].

### Study selection and characteristics

On 27 October 2023, a systemic search was conducted across PubMed, Scopus, and Web of Science to identify randomized controlled trials (RCTs) and prospective studies reporting data on the efficacy of salvage therapies in PCa patients with BCR after RP or RT. The detailed search strategy is shown in Supplementary Appendix 1. Two investigators independently screened titles and abstracts for eligibility, followed by full-text reviews of relevant studies. Manual searches of reference lists of relevant articles were also carried out to find additional studies of interest. Disagreements were resolved by discussion with co-authors.

### Inclusion and exclusion criteria

To formulate our clinical question, we applied the PICO framework [15]. Our study population included patients with PCa experiencing BCR after RP or RT without visible locoregional recurrence or distant metastasis (Patients). We focused on a wide range of therapeutic interventions aiming to improve oncologic outcomes, encompassing various treatment modalities for managing BCR (Interventions), comparing these interventions against the established standard of care (Comparison). The primary outcome of interest was distant metastasis-free survival (MFS). Secondary endpoints included progression-free survival (PFS) and overall survival (OS) (Outcome). PFS includes either BCR or clinical progression, while MFS focuses solely on the absence of metastatic disease. Additionally, the definition of BCR and progression varied across the included studies. These definitions are detailed in Table 1. We excluded retrospective and single-arm studies, reviews, editorial comments, replies to authors, and non-English language articles. Studies involving non-medical compounds were also omitted. Additionally, we excluded studies comparing different radiation therapy techniques, such as dose and fraction schedule, to avoid potential confusion and ensure clearer comparisons across studies.

**Table 1:** Study demographics of included 19 studies.

Study, authorå	Study design	Primary treatment	Definition of BCR and inclusion criteria	Definition of progression	Treatment duration	Treatment 1	Treatment 2	Treatment 3
SALV-ENZA Tran et al. [29]	RCT	RP	PSA ≥ 0.05 ng/ml GS8-10 GS7 with pT3 or R1	PSA ≥ 0.2 ng/ml	6 months	Enzalutamide (160 mg) + RT (66.6–70.2 Gy/37–39 fr)	Placebo+RT (66.6–70.2 Gy/37–39 fr)	NA
FORMULA 509 Nguyen et al. [34]	RCT	RP	PSA ≥ 0.1 ng/ml one or more unfavorable features (GS8-10, PSA > 0.5, pT3/T4, pN1 or radiographic N1, PSADT < 10 months, positive margins, persistent PSA, gross local/regional disease, or Decipher High Risk)	NA	6 months	Abiraterone (1000 mg) + predonisone (5 mg) + Apalutamide (240 mg) + ADT + RT ( ± PLNRT)	Bicalutamide (50 mg) + ADT + RT ( ± PLNRT)	NA
NRG Oncology/RTOG 0534 SPPORT Pollack et al. [37]	RCT	RP	PSA ≥ 0.1 ng/ml pT2/3 GS ≤ 9	PSA ≥ nadir + 2.0 ng/ml local failure metastasis death	ADT: 6 weeks	RT (64.8–70.2 Gy)	RT (64.8–70.2 Gy) + ADT	RT (19.8–25.2 Gy) + ADT + PLNRT (45 Gy)
RTOG 9601 Jackson et al. [54] Shipley et al. [41]	RCT	RP	PSA ≥ 0.2 ng/ml pT2 with positive margin/T3	PSA ≥ nadir + 0.3 ng/ml	2 years	Bicalutamide (150 mg) + RT (64.8 Gy/36 fr)	RT (64.8 Gy/36 fr)	NA
JCOG0401 Yokomizo et al. [24]	RCT	RP	PSA ≥ 0.4 ng/ml pT0/2/3 pN0/x	PSA rise if previous <0.4 ng/ml: ≥0.4 ng/ml PSA rise if previous >0.4 ng/ml: Any increase PSA at any point: >PSA at enrolment	Bicalutamide should be continued as long as it is effective	Bicalutamide (80 mg)	RT (64.8 Gy/36 fr) ± Bicalutamide (80 mg)	NA
GETUG-AFU 16 Carrie et al. [23, 42]	RCT	RP	PSA ≥ 0.2 ng/ml	PSA > nadir + 0.5 ng/ml	ADT: 6 months	RT (66 Gy/33 fr) + ADT	RT (66 Gy/33 fr)	NA
EMBARK Freedland et al. [31]	RCT	RP ± RT or RT	PSA ≥ 2 ng/ml (post-RT) PSA ≥ 1 ng/ml (post-RP) PSADT ≤ 9 months	PSADT ≤ 10 months	36 weeks	Enzalutamide(160 mg) + ADT	Enzalutamide (160 mg)	Placebo + ADT
PRESTO Aggarwal et al. [36]	RCT	RP ± RT	PSA ≥ 0.5 ng/ml PSADT ≤ 9 months	PSA rise to ≥25% PSA ≥ nadir + 2 ng/ml	52 weeks	Apalutamide (240 mg) + Abiraterone (1000 mg) + predonine (10 mg) + ADT	Apalutamide (240 mg) + ADT	ADT
NCT01790126 Aggarwal et al. [26]	RCT	RP ± RT or RT	PSA ≥ 1.0 ng/ml (post-RP) PSA ≥ nadir + 2.0 ng/ml (post-RT) PSADT ≤ 12 months	PSA rise to ≥50% PSA ≥ nadir + 2 ng/ml	12 months	Apalutamide (240 mg) + ADT	Apalutamide (240 mg)	ADT
NCT01786265 Spetsieris et al. [33]	RCT	RP ± RT or RT	PSA ≥ 0.2 ng/ml (post-RP) PSA ≥ nadir + 2.0 ng/ml (post-RT)	PSA ≥ 1.0 ng/ml	8 months	Abiraterone (1000 mg) + predonine (5 mg) + ADT	ADT	NA
NCT01751451 Autio et al. [28]	RCT	RP ± RT	PSA ≥ 1.0 ng/ml	PSA rise to ≥25%	8 months	Abiraterone (1000 mg) + predonine (10 mg) + ADT	Abiraterone (1000 mg) + predonine (10 mg)	ADT
TAX3503 Morris et al. [30]	RCT	RP ± RT	PSA ≥ 1.0 ng/ml PSADT ≤ 9 months	PSA ≥ 0.05 ng/ml	DOC: 10 cycles ADT: 18 months	Docetaxel (75 mg/m^2^) + ADT	ADT	NA
NCT00764166 Oudard et al. [35]	RCT	RP ± RT	PSA ≥ 0.2 ng/ml	PSA ≥ 0.2 ng/ml	DOC: 6 cycles ADT: 12 months	Docetaxel (70 mg/m^2^) + ADT	ADT	NA
TOAD Duchesne et al. [38]	RCT	RP ± RT or RT	PSA ≥ 0.2 ng/ml (post-RP) PSA ≥ nadir + 2.0 ng/ml (post-RT)	Radiological progression	NA	Delayed ADT	Immediate ADT	NA
NCT00928434 Crawford et al. [39]	RCT	RP or other primary therapy	PSA ≥ 0.2 ng/ml (post-RP) 3 separate PSA ≥ nadir PSA (post other primary therapy)	PSA progression additional PCa therapy death	T1: 7 months	Intermittent ADT	Continuous ADT	NA
NCIC Crook et al. [40]	RCT	RT	PSA ≥ 3.0 ng/ml (post RT)	NA	T1: 8 months	Intermittent ADT	Continuous ADT	NA
ARTS Schröder et al. [32]	RCT	RP ± RT, RT	PSA ≥ 0.4 ng/ml (post-RP) PSA ≥ 2.0 ng/ml (post-RT) PSADT 3–24 months cT1-T3aN0M0	PSADT ≤ 3 months PSA > 20 ng/ml (post-RT) PSA > 10 ng/ml (post-RP) PSA rise ≥50% pathological progression radiological progression	2 years	Dutasteride (0.5 mg)	Placebo	NA
PROTECT Beer et al. [27]	RCT	RP ± RT or ±ADT	Increase PSA PSA ≥ 3.0 ng/ml, 1.25 × nadia (previous ADT)	PSA ≥ 3.0 ng/ml	NA	Sipuleuce-T	Placebo	NA
Goluboff et al. [25]	RCT	RP ± RT or ADT	PSA ≥ 0.4 ng/ml PSA rise ≥10%	PSA progression	NA	Exisulind (250 mg × 2/day)	Placebo	NA

*ADT* androgen deprivation therapy, *DOC* docetaxel, *GS* Gleason score, *HDR* high dose-rate brachytherapy, *NA* not available, *PSA* prostate-specific antigen, *PSADT* prostate-specific antigen doubling time, *RCT* randomized controlled trial, *RP* radical prostatectomy, *RT* radiation therapy.

### Data extraction

Two authors independently extracted details on study design, patient characteristics, inclusion criteria, definition of disease progression, oncologic outcomes, and adverse events (AEs). Subsequently, the results of the Kaplan–Meier analyses, hazard ratios (HRs) with 95% confidence intervals (CIs) from Cox regression models for PFS, MFS, and OS were retrieved. Studies providing HR data with detailed statistical measures were included in the meta-analysis and NMA. Studies lacking such detailed data were considered for the systematic review but excluded from the meta-analysis and NMA. Discrepancies were resolved by consensus with co-authors.

### Risk-of-bias assessment

Study quality and risk of bias were assessed using the Risk-of-Bias version 2 (ROB2) tool as outlined in the Cochrane Handbook for Systematic Reviews of Interventions (Supplementary Fig. 1) [16]. The presence of confounders was determined by consensus and a review of the literature. The Risk-of-Bias assessments of each study were conducted independently by two authors.

### Statistical analyses

#### Standard pairwise meta-analysis

Quantitative data synthesis was carried out with the R statistical software 4.3.0 (R Foundation for Statistical Computing, Vienna, Austria). For our calculations, we followed the methods recommended by the working group of the Cochrane Collaboration [17]. Based on the likely heterogeneity across studies, a random-effect model was used for calculations of HRs [18]. To assess and compare the MFS and OS of different treatments for BCR, we calculated pooled HRs with 95% CI using the “meta” package in R. We utilized forest plots to visualize event rates and effect measures. In our analysis, the statistical significance level was set at *p* < 0.05. The minimum number of studies to perform a meta-analysis was two. We assessed heterogeneity using Cochran’s Q test and explored its causes when significant (*p* < 0.05) [19]. To evaluate the presence of publication bias, funnel plots were used (Supplementary Fig. 2). We performed Egger’s test if 10 or more studies were included in each analysis.

#### Network meta-analysis

A network meta-analysis (NMA) using random-effect models with a frequentist approach was carried out for direct and indirect treatment comparisons [20, 21]. In the assessment of oncological outcomes, contrast-based analyses were applied with estimated differences in the log HR and the standard error calculated from the published HR and 95% CIs [22]. When a three-arm trial reported only two comparisons, we calculated the additional comparison independently. The relative effects were presented as HRs and 95% CIs [21]. We also estimated the relative ranking of the different treatments for each outcome using the surface under the cumulative ranking (SUCRA) [21]. Network plots were utilized to illustrate the connectivity of the treatment networks (Supplementary Fig. 3). All statistical analyses were performed using R version 4.3.0 (R Foundation for Statistical Computing, Vienna, Austria), utilizing the “netmeta” package in R.

## Results

### Study selection and characteristics

**Fig. 1:**
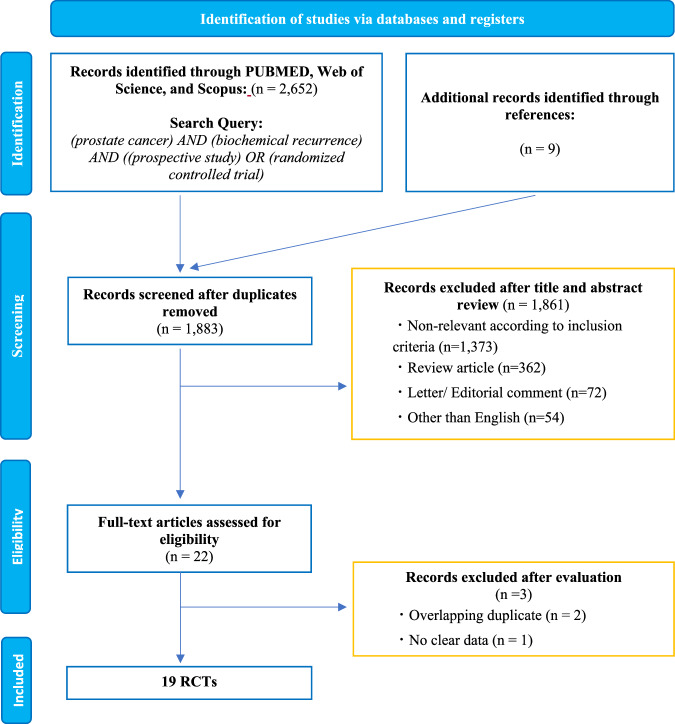
PRISMA flowchart illustrating the article selection process. The flowchart demonstrates the systematic process according to the PRISMA guidelines.

The search string is presented in Fig. 1. According to the application of our inclusion and exclusion criteria, a total of 19 RCTs [23–41], comprising 9117 PCa patients with BCR following definitive local treatments, were selected. The patient characteristics and their outcomes are detailed in Tables 1 and 2. Eighteen studies included patients who had undergone RP [23–39, 41]. Of these, 11 studies [25–28, 30–33, 35, 36, 38] allowed the use of adjuvant or salvage RT after RP. Seven studies [26, 31–33, 38–40] included RT as the primary treatment. In the context of RT-based strategies, most studies investigated the effectiveness of combining RT with hormone therapy: two on ARSIs [29, 34], two on bicalutamide (BIC) [24, 41], and two on ADT [37, 42], with one study [37] also examining elective pelvic node irradiation. In terms of hormone-based therapies, five studies [26, 28, 31, 33, 36] used the addition of ARSIs, and two studies [30, 35] combined DOC with ADT as a primary intervention. Three studies [38–40] investigated the timing and duration of ADT. The other three studies used dutasteride, sipuleucel-T, or exisulind. The definition of BCR and progression varied among studies. The PROTECT trial [27] defined BCR as an increase in prostate-specific antigen (PSA) without a specific threshold, whereas others set different PSA level criteria (for post-RP: PSA levels above 0.05–1.0 ng/ml; for post-RT: PSA levels above 1.0–2.0 ng/ml or above nadir + 2.0 ng/ml). Notably, none of the studies utilized PSMA-PET for metastasis detection; all used conventional imaging modalities such as CT, bone scan, and MRI. The median follow-up period ranged from 30 to 112 months.

**Table 2:** Patient characteristics of included 19 studies.

Study, author	No. of patients	Median age	PSA	Median follow-up, mo
SALV-ENZA Tran et al. [29]	Total: 86 ENZ + RT: 43 RT: 43	ENZ + RT: 69 (51–82) RT: 66 (52–81)	ENZ + RT: 0.3 (0.06–2.6) RT: 0.3 (0.07–4.6)	34 (0–52)
FORMULA 509 Nguyen et al. [34]	Total: 345 ABI + APA + ADT + RT: 173 BIC + ADT + RT: 172	NA	NA	34 (6–53)
NRG Oncology/RTOG 0534 SPPORT Pollack et al. [37]	Total: 1716 RT: 564 RT + ADT: 578 RT + ADT + PLNRT: 574	RT: 64 (42–84) RT + ADT: 64 (39–80) RT + ADT + PLNRT: 64 (44–80)	RT: 0.32 (0.1–1.96) RT + ADT: 0.40 (0.1–1.93) RT + ADT + PLNRT: 0.32 (0.1–1.93)	98.4 (79.2–112.8)
RTOG 9601 Jackson et al. [54] Shipley et al. [41]	Total: 760 BIC + RT: 384 RT: 376	BIC + RT: 65 (59–69) RT: 65 (60–69)	BIC + RT: 0.6 (0.2–1.0) RT: 0.6 (0.2–1.2)	156
JCOG0401 Yokomizo et al. [24]	Total: 210 BIC: 105 RT + BIC: 105	BIC: 70 (65–74) RT + BIC: 71 (67–75)	BIC: 0.47 (0.43–0.55) RT + BIC: 0.48 (0.43–0.57)	66.0 (34.8–90.0)
GETUG-AFU 16 Carrie et al. [23, 42]	Total: 742 RT + ADT: 369 ADT: 373	RT + ADT: 69.5 (62.9–72.1) RT: 66.8 (61.5–71.9)	RT + ADT: 0.3 (0.2–0.5) RT: 0.3 (0.2–0.5)	112 (102–123)
EMBARK Freedland et al. [31]	Total: 1068 ENZ + ADT: 355 RP alone: 90 (25.4) RT alone: 86 (24.2) RP + RT: 179 (50.4) ENZ: 355 RP alone: 99 (27.9) RT alone: 90 (25.4) RP + RT: 166 (46.8) ADT: 358 RP alone: 75 (20.9) RT alone: 104 (29.1) RP + RT: 179 (50.0)	ENZ + ADT: 69 (51–87) ENZ: 69 (49–93) ADT: 70 (50–92)	ENZ + ADT: 5.0 (1.0–308.3) ENZ: 5.3 (1.1–37.0) ADT: 5.5 (1.1–163.3)	60.7
PRESTO Aggarwal et al. [36]	Total: 504 APA + ABI + ADT: 169 APA + ADT: 168 ADT: 167	NA	NA	NA
NCT01790126 Aggarwal et al. [26]	Total: 90 APA + ADT: 31 RP alone: 10 (32.3) RT alone: 4 (12.9) RP + RT: 18 (58.1) APA: 29 RP alone: 6 (20.7) RT alone: 6 (20.7) RP + RT: 17 (58.6) ADT: 30 RP alone: 3 (0.1) RT alone: 5 (16.7) RP + RT: 21 (70.0)	APA + ADT: 67.0 (54–78) APA: 66.0 (55–79) ADT: 68.5 (46–80)	APA + ADT: 4.1 (1.2–38.8) APA: 2.7 (1.0–42.3) ADT: 4.0 (1.2–29.8)	NA
NCT01786265 Spetsieris et al. [33]	Total: 197 ABI + ADT: 99 RP: 93 (94) RP + RT: 48 (48) RT alone: 6 (6) ADT: 98 RP: 93 (95) RP + RT: 49 (50) RT alone: 5 (5)	ABI + ADT: 65 (44–80) ADT: 65 (42–85)	ABI + ADT: 1.2 (0.2–11.1) ADT: 1.0 (0.2–33.3)	64.4 (40.7–90.3)
NCT01751451 Autio et al. [28]	Total: 120 ABI + ADT: 41 salvage RT: 24 (59) ABI: 37 salvage RT: 23 (59) ADT: 42 salvage RT: 27 (64)	ABI + ADT: 65 (53–74) ABI: 64 (43–83) ADT: 66 (46–78)	ABI + ADT: 5.8 (1.2–45.1) ABI: 3.1 (1.2–35.4) ADT: 4.1 (1.0–48.3)	NA
TAX3503 Morris et al. [30]	Total: 413 DOC + ADT: 207 postoperative RT: 65 (31.4) ADT: 206 postoperative RT: 76 (36.9)	DOC + ADT: 66 (60–71) ADT: 65 (60–69)	DOC + ADT: 0.8 (0.5–1.4) ADT: 0.7 (0.5–1.7)	33.6
NCT00764166 Oudard et al. [35]	Total: 250 DOC + ADT: 125 salvage RT: 54 (43.2) ADT: 125 salvage RT: 56 (44.8)	DOC + ADT: 64 (58–70) ADT: 66 (61–71)	DOC + ADT: 2.6 (1.0–6.2) ADT: 2.9 (1.0–6.0)	30
TOAD Duchesne et al. [38]	Total: 261 Delayed ADT: 137 RT: 88 (64) RP ± RT: 49 (36) Immediate ADT: 124 RT: 77 (62) RP ± RT: 47 (38)	Delayed ADT: 70.0 (IQR: 50.7–85.0) Immediate ADT: 71.1 (IQR: 54.0–88.0)	NA	5 years (IQR: 3.3–6.2)
NCT00928434 Crawford et al. [39]	Total: 403 Intermittent ADT: 175 RP: 39 (22) RT: 107 (61) Cryotherapy: 24 (14) Other: 5 (3) Not recorded: 0 Continuous ADT: 228 RP: 48 (21) RT: 153 (67) Cryotherapy: 23 (10) Other: 3 (1) Not recorded: 1 (<1)	Intermittent ADT: 73 (50–91) Continuous ADT: 71 (51–89)	Intermittent ADT: 5.15 (0.2–655) Continuous ADT: 4.96 (0.17–262)	NA
NCIC Crook et al. [40]	Total: 1386 Intermittent ADT: 690 Continuous ADT: 696	Intermittent ADT: 74.2 (range: 29.4–89.7) Continuous ADT: 74.4 (range: 45.3–88.9)	Intermittent ADT 3–15: 531 (77.0) >15: 159 (23.0) Continuous ADT 3–15: 535 (76.9) >15: 160 (23.0) Missing: 1 (0.1)	6.9 years (range: 2.8–11.2)
ARTS Schröder et al. [32]	Total: 294 Dutasteride: 147 RP alone: 91 (61.9) RT alone: 33 (22) RP + RT: 23 (15.6) Placebo: 147 RP alone: 90 (61.2) RT alone: 28 (19) RP + RT: 29 (19.7)	mean age ± SD Dutasteride: 69.7 ± 5.76 Placebo: 68.6 ± 6.53	NA	Dutasteride 722 days Placebo: 456 days
PROTECT Beer et al. [27]	Total: 176 Sipuleuce-T: 117 adjuvant HT: 21 (17.9) adjuvant RT: 20 (17.1) salvage RT: 52 (44.4) Placebo: 59 adjuvant HT: 9 (15.3) adjuvant RT: 8 (13.6) salvage RT: 28 (47.5)	Sipuleuce-T: 64 (48–79) Placebo: 67 (47–78)	Sipuleuce-T: 2.3 (0.8–33.0) Placebo: 2.3 (0.8–20.5)	NA
Goluboff et al. [25]	Total: 96 Exisulind: 47 Placebo: 49	Exisulind: 67.6 (48–87) Placebo: 65.8 (51–78)	Exisulind: 1.75 Placebo: 1.7	NA

*ADT* androgen deprivation therapy, *ABI* abiraterone, *APA* apalutamide, *BT* brachytherapy, *DOC* docetaxel, *ENZ* enzalutamide, *NA* not available, *PSA* prostate-specific antigen, *RP* radical prostatectomy, *RT* radiation therapy.

Due to the heterogeneity among the definitions of PFS, we conducted only a qualitative synthesis of the data. Therefore, studies providing only PFS data were excluded from our analyses. Additionally, among the studies reviewed, two trials [24, 34] exhibited unique designs that led to their exclusion from our NMA. In the JCOG0401 trial [24], which compared BIC with RT, approximately half of the patients in the RT arm received BIC after randomization. Similarly, the FORMULA 509 trial [34] allowed pelvic lymph node radiation therapy (PLNRT) for patients with pN1 and offered it as an option for those with pN0. These unique design elements made it challenging to integrate their results into the NMA framework.

### Risk-of-bias assessment

The results of bias evaluation for each domain across the included studies are presented in Supplementary Fig. 1. Most RCTs exhibited a low risk of bias across the majority of domains. However, some concerns were identified in certain areas for a few studies. Funnel plots of each analysis are depicted in Supplementary Fig. 2.

### RT-based treatment strategies for patients with BCR after RP

A total of six RCTs, comprising 3859 participants, evaluated RT-based treatments for patients with BCR after RP [23, 24, 29, 34, 37, 41]. These studies administered single-agent or combined hormone therapy (HT), such as enzalutamide (ENZ), abiraterone (ABI), apalutamide (APA), bicalutamide (BIC), and ADT administered between 6 weeks to 2 years in combination with RT. Detailed descriptions of the studies can be found in Tables 1 and 2.

The SALV-ENZ trial [29] showed that adding ENZ to RT improved freedom from PSA progression (HR: 0.42, 95% CI: 0.19–0.92, *p* = 0.031), especially for high-risk patients, such as pT3 (HR: 0.22, 95% CI: 0.07–0.69) and surgical margin-positive (HR: 0.14, 95% CI: 0.03–0.64). The JCOG0401 trial [24] demonstrated RT with/without BIC prolonged PFS compared to BIC alone (HR: 0.56, 95% CI: 0.38–0.82, *p* = 0.001). The FORMULA 509 trial [34] compared ABI + APA with BIC, both added to ADT and RT. No significant difference was noted overall; however, a subgroup analysis with PSA > 0.5 ng/ml revealed a significant improvement in both PFS (HR: 0.50, 90% CI: 0.30–0.86) with ABI + APA. The NRG Oncology/RTOG 0534 SPPORT trial [37] demonstrated the benefits of adding PLNRT to RT and ADT in freedom from progression (HR: 0.82, 95% CI: 0.63–1.07, *p* = 0.048). However, this was accompanied by an increase in the incidence of acute AEs Grade 2+ (RT + ADT: 37.7%, PLNRT + RT + ADT: 44.6%).

#### Standard pairwise meta-analysis (HT + RT vs. RT alone)

**Fig. 2:**
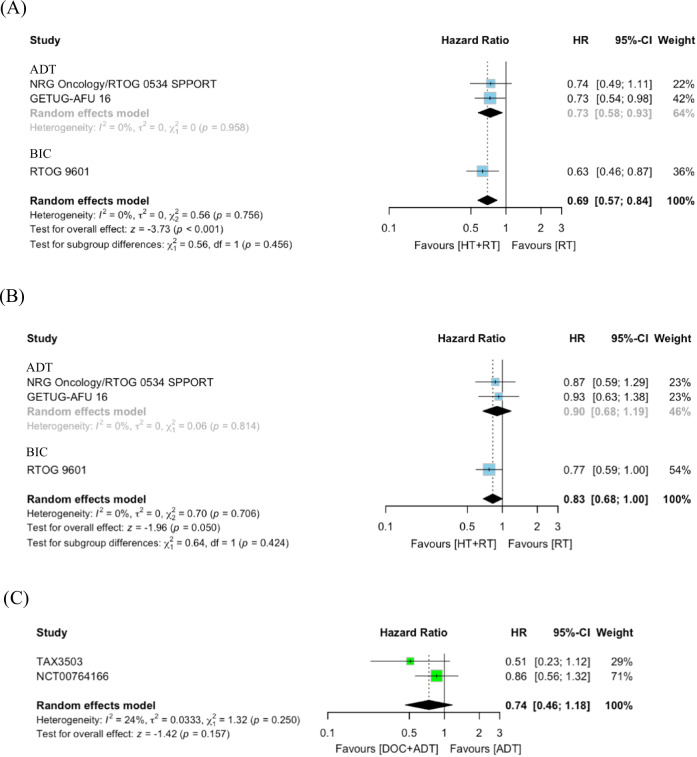
Forest plot of the meta-analysis comparing different treatment modalities. **A** MFS for HT + RT vs. RT: this panel shows the meta-analysis results for MFS comparing HT combined with RT versus RT alone. **B** OS for HT + RT vs. RT: this panel illustrates OS outcomes for HT + RT compared to RT alone. **C** OS for DOC + ADT vs. ADT: this panel demonstrates OS comparing DOC combined with ADT versus ADT alone. Symbols represent HRs with 95% confidence intervals (CIs), and the diamonds indicate pooled estimates. OS overall survival, RT radiotherapy, HT hormone therapy, MFS metastasis-free survival, DOC docetaxel, CI confidence interval, ADT androgen deprivation therapy, HR hazard ratio.

In our pairwise meta-analyses, we were able to summarize data from three articles [23, 37, 41]. Combining HT with RT was found to significantly improve MFS compared to RT alone (HR: 0.69, 95% CI: 0.57–0.84, *p* < 0.001). There was some evidence for improved OS, which did not reach conventional levels of statistical significance (HR: 0.83, 95% CI: 0.68–1.00, *p* = 0.05) (Fig. 2). Cochran’s Q test revealed no significant heterogeneity among the included studies.

#### Network meta-analysis (NMA)

**Fig. 3:**
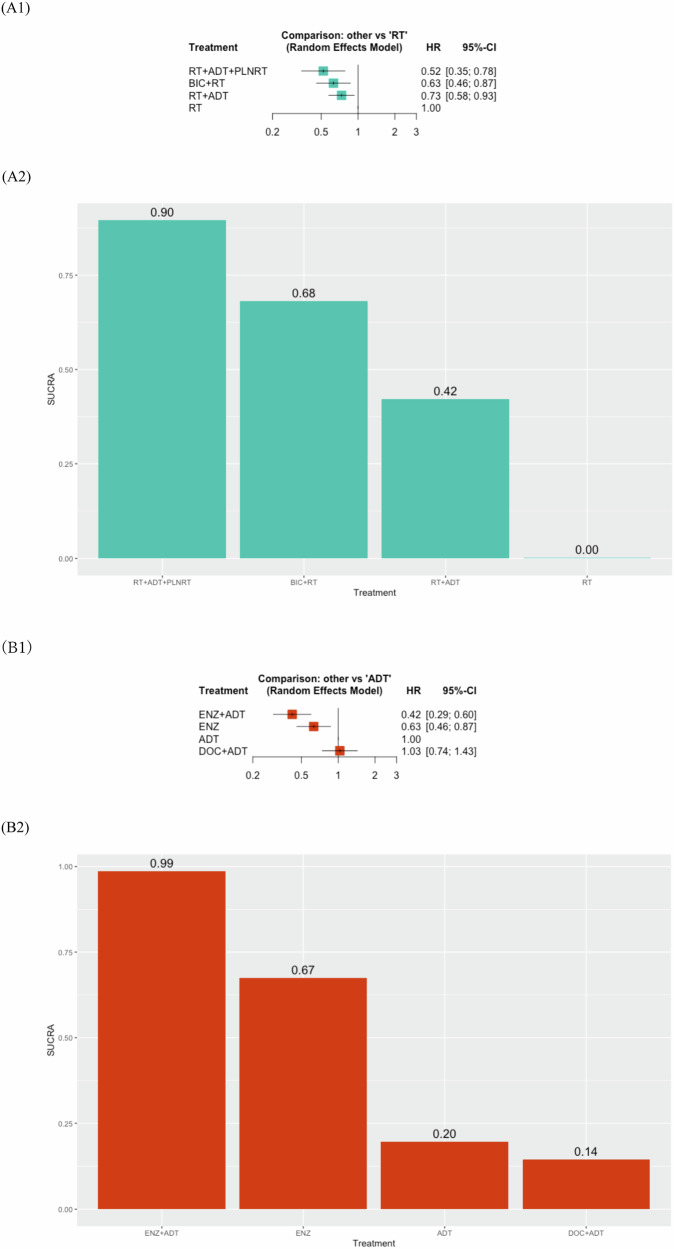
Results of NMAs for MFS of patients with BCR. **A** RT-based treatment: **A1** Forest plots (RT as a comparator), **A2** Treatment ranking based on SUCRA graph, **B** Hormone-based treatment: **B1** Forest plots, **B2** Treatment ranking based on SUCRA graph.

**Fig. 4:**
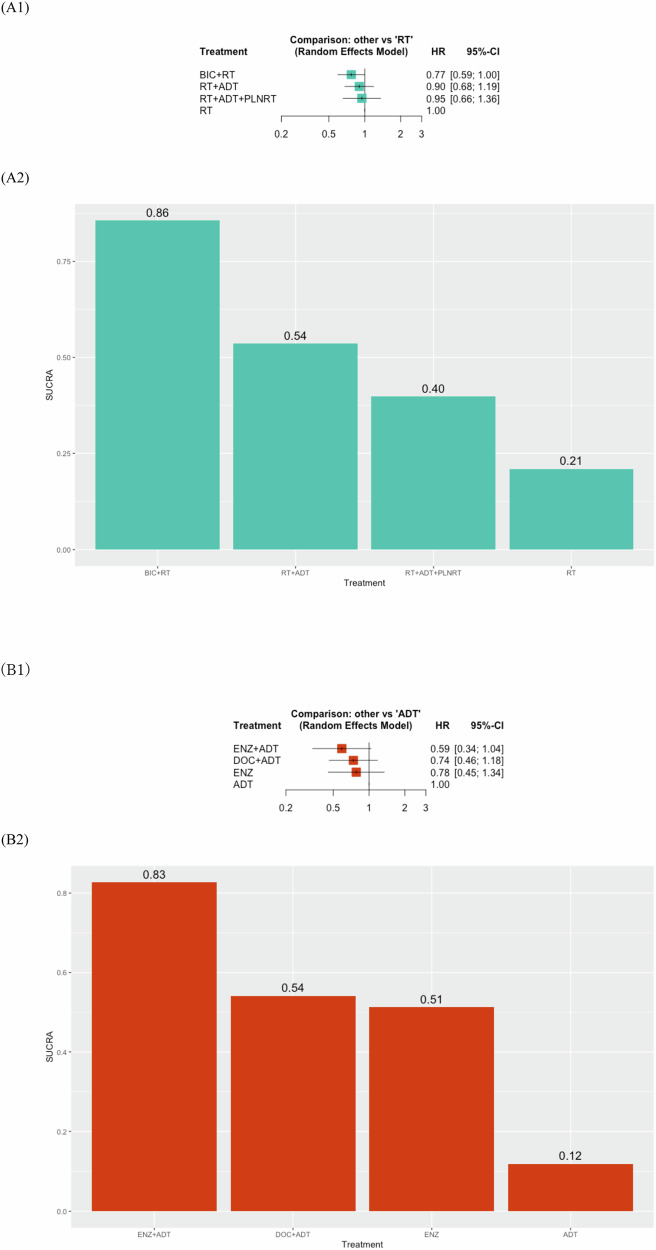
Results of NMAs for OS of patients with BCR. **A** RT-based treatment: **A1** Forest plots, **A2** Treatment ranking based on SUCRA graph, **B** Hormone-based treatment: **B1** Forest plots, **B2** Treatment ranking based on SUCRA graph. PRISMA preferred reporting items for systematic reviews and meta-analyses, MFS metastatic-free survival, HT hormone therapy, RT radiation therapy, OS overall survival, DOC docetaxel, ADT androgen deprivation therapy, NMA network meta-analysis, BCR biochemical recurrence, SUCRA surface under the cumulative ranking.

Our NMAs included three RCTs with four RT-based treatments [23, 37, 41]. The RT-based NMA focused on the addition of HT or PLNRT to RT. All combinations, including ADT + RT (HR: 0.73, 95% CI: 0.58–0.93), BIC + RT (HR: 0.63, 95% CI: 0.46–0.87), and PLNRT + ADT + RT (HR: 0.52, 95% CI: 0.35–0.78), significantly improved MFS compared to RT alone as shown in Fig. 3. However, compared to RT + ADT, no treatment combination demonstrated significant improvement in MFS (Supplementary Fig. 4). Based on SUCRA analysis, the combination of RT + ADT + PLNRT (90%) had the highest likelihood of providing the maximal MFS benefit, followed by BIC + RT (68%) and RT + ADT (42%). In terms of OS, no treatment combination showed significant improvement (Fig. 4). According to SUCRA analysis for OS, BIC + RT (86%) ranked highest for OS benefit. No significant heterogeneity was observed in each analysis.

### Hormone-based treatment strategies for patients with BCR after RP or RT

#### Androgen receptor signaling inhibitors (ARSIs)

Five RCTs comprising 1979 participants assessed the impact of ARSIs administered for 8–12 months, on oncological outcomes in patients with BCR after definitive local treatment [26, 28, 31, 33, 36].

The EMBARK trial [31] showed that ENZ + ADT improved both PFS (HR: 0.07, 95% CI: 0.03–0.14, *p* < 0.001) and MFS (HR: 0.42, 95% CI: 0.30–0.61, *p* < 0.001). APA + ADT improved PSA-PFS in the PRESTO trial (HR: 0.52, 95% CI: 0.35–0.77), but not in the NCT01790126 trial (0.56, 95% CI: 0.23–1.36, *p* = 0.196) (Supplementary Table 2). The combination of ABI and ADT demonstrated effectiveness in reducing disease progression in the NCT01786265 trial [33] (HR: 0.64, 95% CI: 0.47–0.87, *p* = 0.004); however, no significant difference in median PSA-PFS was found in the NCT01751451 study [28] (ABI + ADT: 64.4 weeks, 95% CI: 57.9–NA; ADT: 54.9 weeks, 95% CI: 47.9–60.7 weeks). Furthermore, the combination of ABI, APA, and ADT showed improvements in PFS compared to ADT alone in the PRESTO trial [36] (HR: 0.48, 95% CI: 0.32–0.71). The AE profiles are similar to those observed in previous RCTs for each respective drug [43–46].

#### Docetaxel (DOC)

Two RCTs comprising 663 participants assessed the impact of docetaxel (DOC) on oncological outcomes in patients with BCR post-RP [30, 35]. The duration of treatment varied among studies, with DOC ranging from 6 to 10 cycles and ADT from 12 to 18 months. DOC use was not significantly associated with PFS due to the wide range of CIs in two studies. In addition, DOC use was associated with a higher incidence of neutropenia, alopecia, and fatigue, among others.

#### Standard pairwise meta-analysis (DOC + ADT vs. ADT alone)

Adding DOC to ADT did not significantly improve OS compared to ADT alone (HR: 0.74, 95% CI: 0.46–1.18, *p* = 0.2) (Fig. 2). As the NCT00764166 trial [35] was the only study to evaluate MFS, a meta-analysis for MFS was not performed. Cochran’s Q test revealed no significant heterogeneity among the studies included.

#### Timing and duration of ADT

The NCIC trial [40] conducted a comparison between intermittent ADT (*n* = 690) to continuous ADT (*n* = 696). Intermittent ADT was associated with significantly improved outcomes for hot flashes (*p* < 0.001), desire for sexual activity (*p* < 0.001), and urinary symptoms (*p* < 0.01), while no significant difference was shown in OS. In the NCT00928434 trial [39], which randomized patients experiencing BCR to either intermittent ADT (*n* = 175) or continuous ADT (*n* = 228), the sexual drive was significantly improved in patients undergoing intermittent ADT compared to those receiving continuous ADT (*p* = 0.027).

The TOAD trial [38] assessed the implication of initiating ADT on a delayed basis (*n* = 137) compared to immediate initiation of ADT (*n* = 124). There was no significant difference in OS (HR: 0.59, 95% CI: 0.26–1.30, *p* = 0.19). Regarding QoL between the two arms, arm-specific rates of change over time did not statistically differ (*p*_interaction_ = 0.14).

#### Network meta-analysis of hormone-based treatments

As shown in Fig. 3, ENZ + ADT (HR: 0.42, 95% CI: 0.29–0.60) and ENZ alone (HR: 0.63, 95% CI: 0.46–0.87) significantly improved MFS compared to ADT alone. SUCRA analysis ranked ENZ + ADT (99%) as the most effective for MFS, followed by ENZ alone (67%). In terms of OS, no agents significantly improved MFS compared to ADT alone (Fig. 4). Based on the SUCRA analysis, ENZ + ADT (83%) had the highest likelihood of providing the maximal benefit for OS. Cochran’s Q test revealed no significant heterogeneity in each analysis.

### Other treatments

Three RCTs, comprising 566 patients, assessed the impact of other agents on oncological outcomes in patients with BCR. All of these studies used a placebo as a comparator; therefore, we did not include them in our NMAs. Dutasteride significantly reduced disease progression (risk ratio [RR]): 0.41, 95% CI: 0.25–0.67, *p* < 0.001 in the ARTS trial [32], while no significant difference in PFS and MFS were noted with sipuleucel-T in the PROTECT trial [27]. Goluboff et al. [25] demonstrated that exisulind showed potential benefits in high-risk patients. Detailed outcomes and AEs are summarized in Supplementary Table 2.

## Discussion

This systematic review, pairwise meta-analysis, and NMA represent the comprehensive assessment of various interventions on oncological outcomes in PCa patients who experienced BCR following definitive local treatment. Our study highlights several key findings. First, the addition of HT to RT improved MFS compared to RT alone in patients with BCR after RP. Furthermore, our treatment ranking analysis identified PLNRT in combination with ADT and RT to exhibit the highest benefit in terms of MFS. Second, there is evidence suggesting that ARSI-based treatments might improve PFS in patients with BCR following RP or RT compared to ADT alone. Conversely, the impact of DOC on PFS, MFS, and OS appears to be less clear. In addition, ENZ + ADT was shown to be the most effective in enhancing both MFS and OS among hormone-based treatment strategies. Third, although no significant differences were found in PFS and MFS among the overall patient cohort, the addition of ARSI to RT was associated with improvement in PFS and MFS, especially in patients with adverse prognostic factors, such as pT3 staging, positive surgical margins, and elevated PSA levels.

Our meta-analysis indicated that adding HT to RT potentially reduced the risk of metastasis by 31% in PCa patients with BCR after RP, with a suggested but not statistically significant 17% improvement in OS. Our SUCRA analysis revealed PLNRT + ADT + RT as the most effective combination for improving MFS. However, the addition of PLNRT did not demonstrate a statistically significant superiority over ADT + RT in our NMA model either in terms of MFS and OS. Although it is crucial to weigh the benefit against potential AEs, the NRG Oncology/RTOG 0534 SPPORT trial [37] revealed that the addition of PLNRT resulted in increased rates of acute AEs. Interestingly, the addition of BIC to RT was also found to carry great potential in improving MFS and OS compared to RT alone, though the improvement in OS did not reach conventional levels of statistical significance. Although BIC is not recommended for many indications, considering its acceptable AE profile and our results it could potentially be a promising combination partner with RT in BCR after RP; especially those with less aggressive PCa and longer PSA doubling time. It should be noted that the observed benefits of PLNRT and BIC in our analyses were based solely on data from a single study for each: the NRG Oncology/RTOG 0534 SPPORT [37] and the RTOG 9601 trial [47], respectively. This limited data source might lead to over- or under-interpretation. Therefore, more prospective data is needed to further assess the potential benefits of adding PLNRT or BIC to RT.

We found that combining ARSI with ADT reduced the risk of disease progression in the majority of studies, while DOC + ADT did not significantly impact PFS or OS. Our treatment rankings indicated that ENZ + ADT was the most likely to improve MFS compared to ADT alone among hormone-based treatments. Determination of when and for how long ADT should be initiated in the salvage setting remains unclear. For instance, no studies have indicated that immediate ADT significantly improves OS [38, 48]. Furthermore, long-term ADT can lead to various AEs (e.g., cardiovascular events, osteoporosis, cognitive impairment) [49–51]. Additionally, EAU low-risk BCR significantly correlates with more favorable mortality outcomes than high-risk [52]. Therefore, immediate ADT is not recommended for patients with low-risk BCR by guidelines [53]. Considering these findings, alternative treatments that improve mortality within tolerable AEs are desirable for patients with BCR after RT. In the EMBARK trial [31], ENZ + ADT improved in MFS, a strong surrogate for OS in men receiving RT [54], compared to ADT alone. Importantly, this improvement came with a similar frequency of AEs as seen with ADT alone, suggesting that ENZ + ADT could be an effective and safe strategy for patients with BCR following RT. On the other hand, while RT-based treatment is currently the standard therapy for BCR patients after RP [53], the efficacy of hormone-based treatment for these patients remains unclear. In this study, direct comparisons between ADT- and RT-based strategies were not feasible due to the diversity of primary treatments (e.g., RP, RP + adjuvant/salvage RT, RT). Although interpretations should be made with caution due to the short follow-up period and small sample size, the JCOG0401 trial, which compared BIC alone to RT with/without BIC, showed no significant difference in MFS and OS. While RT remains a standard option, ARSIs + ADT may have the potential to become a prime option comparable to RT with/without ADT. However, these discussions have not taken into account the presence of undetectable metastasis by conventional imaging. Recently, PSMA-PET has shown promising effectiveness in detecting metastasis missed by conventional imaging, such as pelvic lymph node metastases (42%) and bone metastases (15%) [55, 56]. Although metastatic-directed therapies (MDT) targeting oligometastases have gained attention, it remains an experimental approach [57]. Therefore, with the future integration of PSMA-PET, treatment decisions are expected to shift from relying on risk stratification or primary treatment to PSMA-PET findings. RCTs comparing PSMA-PET-based MDT and ARSI + RT are eagerly awaited.

Based on our systematic review, the benefit of adding ARSIs to RT was observed, especially for PCa patients with prognostically adverse factors. The SALV-ENZA trial [29] demonstrated that ENZ + RT decreased disease progression compared to RT alone by 58%, particularly in high-risk patients, such as pT3 (78%) and margin-positive (86%). Furthermore, the FORMULA trial [34] also found a significant reduction in disease progression (50%) and metastasis (68%) with the addition of ABI and APA to ADT + RT in patients with PSA > 0.5 ng/ml. In contrast, other studies investigating the effect of ADT [23, 37] and BIC [41] did not demonstrate such a differential benefit in similar settings. Therefore, combining ARSIs with RT could become a key approach in the treatment, especially for PCa patients with these prognostically adverse factors, such as high PSA, pT3, and margin-positive. The results of ongoing studies on ARSIs, such as the BALANCE trial (APA + RT vs. placebo + RT alone) and the STEEL trial [58] (ENZ + ADT + RT vs. ADT + RT) are anticipated to further validate the ARSI + RT combination in the BCR setting.

Our study has several limitations. First, there were notable variations in patient baseline characteristics across the included studies. Additionally, the difference among definitions of BCR and disease progression across studies could lead to heterogeneity in the results and their interpretation. Therefore, due to these inconsistencies, especially regarding the crucial aspect of disease progression in clinical practice, we refrained from conducting a meta-analysis and NMA for this outcome. Furthermore, due to the heterogeneity and the lack of subgroup analysis in the included studies, we were unable to conduct subgroup analyses stratified by patient characteristics. This limitation means we could not determine the optimal treatment for each patient. Second, the duration of follow-up and medication use varied among studies, potentially impacting the assessment of oncological outcomes. Third, the included studies exhibited a diversity of initial treatments, including RP, RT, and RP plus adjuvant/salvage RT. Furthermore, the proportion of patients undergoing these treatments varied among the studies. This mixture of treatment scenarios hindered our ability to conduct distinct analyses for each specific context of salvage therapy following different local treatments. Fourth, the assessment of metastasis in the included studies was based on conventional imaging, and PSMA-PET was not utilized. This raises the possibility of inappropriate treatment for patients with micrometastases due to less sensitive imaging. Fifth, due to the variability in the reporting styles of AEs across the included studies, we were limited to performing meta-analyses or NMAs of AEs. Sixth, the STAMPEDE trial (arm A, J) [59] also investigated patients with BCR and locally advanced PCa; however, separate data for these groups could not be obtained. Thus, we did not include the STAMPEDE trial [59] in our study. Finally, our findings may not fully apply to patients with low-risk BCR, particularly those with a life expectancy of less than 10 years or those unwilling to undergo salvage therapy. For those patients, active follow-up could be a more appropriate management strategy [4].

## Conclusion

We found that combining HT with RT effectively prevents disease progression and metastasis in PCa patients who experienced BCR following definitive local treatment. Furthermore, adding PLNRT to this combination improved MFS. ARSIs improved oncological outcomes when combined with RT or ADT. Further, well-designed RCTs are awaited to clarify the comparative oncologic outcomes of RT-based and hormone-based treatments in different clinical scenarios, with a particular focus on the role of ARSIs.

## Supplementary information

Supplemental Material
