# Supplementary material for: Salvage therapies for biochemical recurrence after definitive local treatment: a systematic review, meta-analysis, and network meta-analysis
Source: Prostate Cancer Prostatic Dis. 2024 Sep 13;28(3):610–22. doi: 10.1038/s41391-024-00890-4 (PMC12399422; doi:10.1038/s41391-024-00890-4)

**Supplementary Information**

**Supplementary Table 1.** PRISMA checklist 2020

**Supplementary Table 2.** Oncological outcomes of included 19 studies

**Supplementary Figure 1.** Risk of bias assessment of the included RCTs

**Supplementary Figure 2.** Funnel plot for publication bias assessment in each analysis

**Supplementary Figure 3.** Network plots of NMA

**Supplementary Figure 4.** NMA results for radiation-based treatment on MFS in patients with BCR: Forest plots with RT + ADT as comparator

**Supplementary Appendix 1**. Search strategy for meta-analysis

**Supplementary Appendix 2**. AMSTAR-2 checklist

**Abbreviations:**

PRISMA = preferred reporting items for systematic reviews and meta-analyses

PPV = positive predictive value

CDR = cancer detection rate

DRE = digital rectal examination

PSA = prostate-specific antigen

ROB2 = risk of bias version 2

ROBINS-I = risk of bias in non-randomized studies of interventions

**Supplementary Table 1.** PRISMA checklist 2020

| **Section and Topic** | **Item #** | **Checklist item** | **Location where item is reported** |
| --- | --- | --- | --- |
| **TITLE** | | |  |
| Title | 1 | Identify the report as a systematic review. | 1 |
| **ABSTRACT** | | |  |
| Abstract | 2 | See the PRISMA 2020 for Abstracts checklist. | 3 |
| **INTRODUCTION** | | |  |
| Rationale | 3 | Describe the rationale for the review in the context of existing knowledge. | 4 |
| Objectives | 4 | Provide an explicit statement of the objective(s) or question(s) the review addresses. | 4 |
| **METHODS** | | |  |
| Eligibility criteria | 5 | Specify the inclusion and exclusion criteria for the review and how studies were grouped for the syntheses. | 5-7 |
| Information sources | 6 | Specify all databases, registers, websites, organisations, reference lists and other sources searched or consulted to identify studies. Specify the date when each source was last searched or consulted. | 5-7 |
| Search strategy | 7 | Present the full search strategies for all databases, registers and websites, including any filters and limits used. | 5-7 |
| Selection process | 8 | Specify the methods used to decide whether a study met the inclusion criteria of the review, including how many reviewers screened each record and each report retrieved, whether they worked independently, and if applicable, details of automation tools used in the process. | 5-7 |
| Data collection process | 9 | Specify the methods used to collect data from reports, including how many reviewers collected data from each report, whether they worked independently, any processes for obtaining or confirming data from study investigators, and if applicable, details of automation tools used in the process. | 5-7 |
| Data items | 10a | List and define all outcomes for which data were sought. Specify whether all results that were compatible with each outcome domain in each study were sought (e.g. for all measures, time points, analyses), and if not, the methods used to decide which results to collect. | 5-7 |
|  | 10b | List and define all other variables for which data were sought (e.g. participant and intervention characteristics, funding sources). Describe any assumptions made about any missing or unclear information. | 5-7 |
| Study risk of bias assessment | 11 | Specify the methods used to assess risk of bias in the included studies, including details of the tool(s) used, how many reviewers assessed each study and whether they worked independently, and if applicable, details of automation tools used in the process. | 5-7 |
| Effect measures | 12 | Specify for each outcome the effect measure(s) (e.g. risk ratio, mean difference) used in the synthesis or presentation of results. | 5-7 |
| Synthesis methods | 13a | Describe the processes used to decide which studies were eligible for each synthesis (e.g. tabulating the study intervention characteristics and comparing against the planned groups for each synthesis (item #5)). | 5-7 |
|  | 13b | Describe any methods required to prepare the data for presentation or synthesis, such as handling of missing summary statistics, or data conversions. | 5-7 |
|  | 13c | Describe any methods used to tabulate or visually display results of individual studies and syntheses. | 5-7 |
|  | 13d | Describe any methods used to synthesize results and provide a rationale for the choice(s). If meta-analysis was performed, describe the model(s), method(s) to identify the presence and extent of statistical heterogeneity, and software package(s) used. | 5-7 |
|  | 13e | Describe any methods used to explore possible causes of heterogeneity among study results (e.g. subgroup analysis, meta-regression). | 5-7 |
|  | 13f | Describe any sensitivity analyses conducted to assess robustness of the synthesized results. | 5-7 |
| Reporting bias assessment | 14 | Describe any methods used to assess risk of bias due to missing results in a synthesis (arising from reporting biases). | 5-7 |
| Certainty assessment | 15 | Describe any methods used to assess certainty (or confidence) in the body of evidence for an outcome. | 5-7 |
| **RESULTS** | | |  |
| Study selection | 16a | Describe the results of the search and selection process, from the number of records identified in the search to the number of studies included in the review, ideally using a flow diagram. | 8-9 |
|  | 16b | Cite studies that might appear to meet the inclusion criteria, but which were excluded, and explain why they were excluded. | 8-9 |
| Study characteristics | 17 | Cite each included study and present its characteristics. | 8-9, Table 1 |
| Risk of bias in studies | 18 | Present assessments of risk of bias for each included study. | 8-9, Supplementary Figure 1 and Supplementary Table 2 |
| Results of individual studies | 19 | For all outcomes, present, for each study: (a) summary statistics for each group (where appropriate) and (b) an effect estimate and its precision (e.g. confidence/credible interval), ideally using structured tables or plots. | 8-9, Table 1 and Table2 |
| Results of syntheses | 20a | For each synthesis, briefly summarise the characteristics and risk of bias among contributing studies. | 8-9, Table 1 |
|  | 20b | Present results of all statistical syntheses conducted. If meta-analysis was done, present for each the summary estimate and its precision (e.g. confidence/credible interval) and measures of statistical heterogeneity. If comparing groups, describe the direction of the effect. | 8-9, Figure 3 |
|  | 20c | Present results of all investigations of possible causes of heterogeneity among study results. | Supplementary figure 3 |
|  | 20d | Present results of all sensitivity analyses conducted to assess the robustness of the synthesized results. | 8-9 |
| Reporting biases | 21 | Present assessments of risk of bias due to missing results (arising from reporting biases) for each synthesis assessed. | Supplementary Figure 1 and Supplementary Table 2 |
| Certainty of evidence | 22 | Present assessments of certainty (or confidence) in the body of evidence for each outcome assessed. | 8-9 |
| **DISCUSSION** | | |  |
| Discussion | 23a | Provide a general interpretation of the results in the context of other evidence. | 10-12 |
|  | 23b | Discuss any limitations of the evidence included in the review. | 10-12 |
|  | 23c | Discuss any limitations of the review processes used. | 10-12 |
|  | 23d | Discuss implications of the results for practice, policy, and future research. | 10-12 |
| **OTHER INFORMATION** | | |  |
| Registration and protocol | 24a | Provide registration information for the review, including register name and registration number, or state that the review was not registered. | 5 |
|  | 24b | Indicate where the review protocol can be accessed, or state that a protocol was not prepared. | NA |
|  | 24c | Describe and explain any amendments to information provided at registration or in the protocol. | NA |
| Support | 25 | Describe sources of financial or non-financial support for the review, and the role of the funders or sponsors in the review. | Authorship form |
| Competing interests | 26 | Declare any competing interests of review authors. | Authorship form |
| Availability of data, code and other materials | 27 | Report which of the following are publicly available and where they can be found: template data collection forms; data extracted from included studies; data used for all analyses; analytic code; any other materials used in the review. | NA |

**Supplementary Table 2.** Oncological outcomes of included 19 studies

| Study, author | Year | Oncological outcome | | | AE | |  |
| --- | --- | --- | --- | --- | --- | --- | --- |
| Radiation based treatment | | | | | | |  |
| SALV-ENZA Tran et al. | 2023 | ENZ Placebo 2yr PFS 84% 66 % p = 0.027  PFS HR: 0.42, 95%CI: 0.19-0.92, p = 0.031 subgroup analyses pT3 vs. pT2  pT3: HR: 0.22, 95% CI: 0.07-0.69 pT2: HR: 1.54, 95% CI: 0.43-5.47, p =0.019 R1 vs R0 R1: HR: 0.14, 95% CI: 0.03-0.64 R0: HR: 1.00, 95% CI: 0.36-2.76, p =0.023 | | | Grade 1 Grade 2  ENZ Placebo ENZ Placebo P-value Fatigue 19 (44.2) 21 (48.8) 9 (20.9) 2 (4.7) 0.38 Diarrhea 13 (30.2) 10 (23.3) 0 1 (2.3) 0.81  Sexuality alteration  10 (23.3) 6 (14.0) 2 (4.7) 3 (7.0) 0.62 Urinary frequency/urgency  14 (32.6) 20 (46.5) 3 (7.0) 1 (2.3) 0.52 | |  |
| FORMULA 509 Nguyen et al. | 2023 | BIC ABI/APA 3yr PFS 68.5% 74.9% p = 0.06 3yr MFS 87.2% 90.6% p = 0.05  PFS HR: 0.71, 90% CI: 0.49-1.03 MFS HR: 0.57, 90% CI: 0.33-1.01 subgroup analysis PSA > 0.5ng/ml PFS: HR: 0.50, 90% CI: 0.30-0.86 MFS: HR: 0.32, 90% CI: 0.15-0.72 | | NA | | | |
| NRG Oncology/RTOG 0534 SPPORT Pollack et al. | 2022 | RT RT+ADT PLNRT+RT+ADT  (Group 1) (Group 2) (Group 3) 5yr PFS 71.1% (66.4-75.9) 82.7% (78.8-86.6) 89.1% (85.9-92.2) Group 3 vs. 1: p <0.0001 Group 2 vs. 1: p<0.0001 Group 3 vs. 2: p = 0.0027  5yr MFS 87.5% (84.7-90.3) 91.4% (89.0-93.7) 91.9% (89.6-94.1) Group 3 vs. 1: p = 0.066 Group 2 vs. 1: p = 0.082 Group 3 vs. 2: p = 0.455  5yr OS 93.5% (91.4-95.6) 96.1% (94.5-97.7) 95.8% (94.2-97.5)  PFS Group 3 vs. 1: HR: 0.50, 95% CI: 0.39-0.64, p <0.0001 Group 2 vs. 1: HR: 0.60, 95% CI: 0.47-0.77, p <0.0001 Group 3 vs. 2: HR: 0.82, 95% CI: 0.63-1.07, p = 0.048  MFS Group 3 vs. 1: 0.52 (0.34-0.81). P =0.00051 Group 2 vs. 1: 0.74 (0.49-1.11), p = 0.047 Group 3 vs. 2: 0.71 (0.45-1.12), p = 0.046  OS Group 3 vs. 1: 0.93 (0.63-2.36). P = 0.332 Group 2 vs. 1: 0.87 (0.59-1.29), p = 0.213 Group 3 vs. 2: 1.07 (0.72-1.58), p = 0.645 | Acute AEs RT RT+ADT PLNRT+RT+ADT All  Grade ≧2 98 (18) 201 (36) 246 (44)  Grade ≧3 18 (3) 41 (7) 63 (11) Blood or bone marrow  Grade ≧2 12 (2) 10 (2) 29 (5)  Grade ≧3 3 (1) 1 (<1) 15 (3) Gastrointestinal  Grade ≧2 11 (2) 22 (4) 38 (7)  Grade ≧3 1 (<1) 5 (1) 4 (1) Renal or genitourinary  Grade ≧2 49 (9) 68 (12) 67 (12)  Grade ≧3 5 (1) 5 (1) 8 (1) Late AEs All  Grade ≧2 308 (57) 322 (58) 350 (62)  Grade ≧3 65 (12) 87 (16) 96 (17) Blood or bone marrow  Grade ≧2 20 (4) 10 (2) 25 (4)  Grade ≧3 3 (1) 2 (<1) 7 (1) Gastrointestinal  Grade ≧2 56 (10) 57 (10) 51 (9)  Grade ≧3 4 (1) 5 (1) 8 (1) Renal or genitourinary  Grade ≧2 202 (37) 194 (35) 223 (40)  Grade ≧3 29 (5) 37 (7) 45 (8) | | |  |  |
| RTOG 9601 Jackson et al. Shipley et al | 2022/2017 | RT+BIC RT 12 yr PFS 56.0% 32.1% 12 yr MFS 85.5% 77.0% 12 yr OS 76.3% 71.3%  PFS HR: 0.48, 95% CI: 0.40-0.58, p <0.001 MFS HR: 0.63, 95% CI: 0.46-0.87, p =0.005 OS HR: 0.77, 95% CI: 0.59-0.99, p = 0.04 | RT RT+BIC  Any grade Grade ≧3 Any grade Grade ≧3 Any AEs 312 (83) 73 (20) 364 (95) 100 (26)  Bladder 225 (60) 26 (7) 232 (61) 27 (7) Bowel 169 (45) 6 (2) 195 (51) 10 (3) | | |  |  |
| JCOG0401 Yokomizo et al. | 2020 | HT RT±HT median PFS (yr) 5.6% (4.5-6.6) 8.6 (7.2-NE)  5 yr PFS 57 (47-66) 70 (60-78) median MFS NR (10.6-NR) NR (NR-NR) 5 yr MFS 94% (87-97) 89% (81-94)  5 yr OS 99% (94-100) 91% (84-95)  PFS HR: 0.56, 95% CI: 0.38-0.82, p = 0.001 MFS HR: 0.90, 95% CI: 0.45-1.81, p = 0.8  OS HR: 1.03, 95% CI: 0.46-2.30, p = 0.9 | HT RT±HT  Any grade Grade ≧3 Any grade Grade ≧3 Gynecomastia 99 (95) 4 (4) 38 (37) 2 (2) Anemia 90 (87) 0 77 (75) 1 (<1) Loss of lipido 86 (83) 0 73 (71) 0 Incontinence 38 (37) 0 65 (63) 5 (5) Thrombocytopenia  47 (45) 0 35 (34) 1 (<1) | | |  |  |
| GETUG-AFU 16 Carrie et al. | 2019/2016 | RT+ADT RT 5 yr PFS 80% (75-84) 62% (57-67) 10 yr PFS 64% (58-69) 49% (43-54) 10 yr MFS 75% (70-80) 69% (63-74) 10 yr OS 86% (81-89) 85% (80-89)  PFS HR: 0.54, 95% CI: 0.43-0.68 MFS HR: 0.73, 95% CI: 0.54-0.98 OS HR: 0.93, 95% CI: 0.63-1.39 | RT RT+ADT  Any grade Grade ≧3 Any grade Grade ≧3 Hot flushes 1(<1) 0 166 (46) 3 (1) Gynecomastia 0 0 4(1) 0 Sweating 0 0 48 (14) 1(<1) Hypertension 1(<1) 0 21 (6) 0 | | |  |  |
| Hormone-based treatment | | | | | |  |  |
| EMBARK Freedland et al. | 2023 | ENZ+ADT ENZ ADT 5yr PFS 97.4% (94.7-98.8) 88.9% (84.6-92.1) 70.0% (64.1-75.1) median MFS  NR (NR-NR) NR (NR-NR) NR (85.1-NR) 5yr MFS 87.3% (83.0-90.6) 80.0% (75.0-84.1) 71.4% (65.7-76.3) 5yr OS 92.2% (88.7-94.7) 89.5% (85.6-92.4) 87.2% (83.0-90.4)  PFS ENZ+ADT vs. ADT HR: 0.07, 95%CI: 0.03-0.14, p <0.001 ENZ vs. ADT HR: 0.33, 95%CI: 0.23-0.49, p <0.001  MFS ENZ+ADT vs. ADT HR: 0.42, 95%CI: 0.30-0.61, p < 0.001  prior RP (± RT) (269 vs 254): HR 0.36, 95% CI: 0.23-0.58  prior only RT (86 vs 104): HR 0.57, 95% CI:0.32-1.00 ENZ vs. ADT HR: 0.63, 95%CI: 0.46-0.87, p = 0.005  OS ENZ+ADT vs. ADT HR: 0.59, 95%CI: 0.38-0.91, p = 0.02 ENZ vs. ADT HR: 0.78, 95%CI: 0.52-1.17, p = 0.23 | ENZ+ADT ADT ENZ  Any Grade Grade ≧3 Any Grade Grade ≧3 Any Grade Grade ≧3 Any AEs 343 (97.2) 164 (46.5) 345 (97.5) 151 (42.7) 347 (98.0) 177 (50.0)  Hot flash 243 (68.8) 2 (0.6) 203 (57.3) 3 (0.8) 77 (21.8) 1 (0.3) Fatigue 151 (42.8) 12 (3.4) 116 (32.8) 5 (1.4) 165 (46.6) 14 (4.0) Arthralgia 97 (27.5) 5 (1.4) 75 (21.2) 1 (0.3) 81 (22.9) 1 (0.3) Hypertension82 (23.2) 2 (0.6) 69 (19.5) 0 67 (18.9) 0 Fall 74 (21.0) 3 (0.8) 51 (14.4) 2 (0.6) 56 (15.8) 5 (1.4)  Back pain 60 (17.0) 1 (0.3) 54 (15.3) 0 62 (17.5) 1 (0.3) | | |  |  |
| PRESTO Aggarwal et al. | 2023 | APA+ABI+ADT APA+ADT ADT median PFS 26.0 months 24.9 months 20.3 months  PFS APA+ABI+ADT vs. ADT HR: 0.48, 95% CI: 0.32-0.71 APA+ADT vs. ADT  HR: 0.52, 95% CI: 0.35-0.77 | NA | | |  |  |
| NCT01790126 Aggarwal et al. | 2022 | APA+ADT APA ADT median PFS 36.1 months 25.8 months 30.9 months PSA progression rate  38.7 % 48.3 % 40.0 %  PFS APA+ADT vs. ADT HR: 0.56, 95% CI: 0.23-1.36, p = 0.196 APA vs. ADT HR: 1.09, 95% CI: 0.49-2.43, p = 0.824 APA+ADT vs. APA HR: 0.40, 95% CI: 0.17-0.98, p = 0.038 | APA+ADT APA ADT Any AEs 31 (100) 29 (100) 28 (96.6) Fatigue 24 (77.4) 19 (65.5) 22 (75.9)  Hot flash 26 (83.9) 9 (31.0) 25 (86.2) Gynecomastia 1 (3.2) 12 (41.4) 3 (10.3) Rash 6 (19.4) 10 (34.5) 3 (10.3)" | | |  |  |
| NCT01786265 Spetsieris et al. | 2021 | ABI+ADT ADT median PFS 27.0 months (23.1-32.0) 19.9 months (19.0-25.5) 1 yr PFS 0.98 (0.95-1.00) 0.88 (0.82-0.95)  PFS HR: 0.64, 95% CI: 0.47-0.87, p = 0.004  RP alone (94): HR: 0.65, 95% CI: 0.42-1.01, p=0.058  RP+EBRT (92): HR: 0.59 95% CI: 0.38-0.93, p=0.023  ERBT alone (11): HR: 1.19, 95% CI: 0.32-4.45, p=0.794 | ABI+ADT ADT  Hot flashes 95 (96) 95 (97) Fatigue 81 (82) 65 (66) Anemia 70 (71) 68 (69) Hypertension 70 (71) 65 (66) | | |  |  |
| NCT01751451 Autio et al. | 2021 | ABI+ADT ABI ADT median PFS 64.4weeks (57.9-NA) 37.5weeks (36.3-44.0) 54.9 weeks (47.9-60.7) | ABI ABI+ADT ADT  Any grade Grade ≧3 Any grade Grade ≧3 Any grade Grade ≧3 Hotflashes 22 (60) 0 33 (80) 0 37(88) 0 Fatigue 17 (46) 0 18 (44) 0 26 (61) 1 (2) Injection site reaction  0 0 0 (24) 0 9 (21) 0 ALT increased 8 (21) 2(5) 7 (17) 2 (5) 4 (10) 0 Hypertension 7 (19) 1 (3) 8 (20) 2 (5) 3 (6) 1 (2) AST increased 9 (25) 1 (3) 6 (14) 1 (2) 3 (7) 0 | | |  |  |
| TAX3503 Morris et al. | 2021 | DOC+ADT ADT median PFS 26.3 months 24.7 months median OS NR NR  PFS HR: 0.81, 95% CI: 0.64-1.02, p = 0.07 OS HR: 0.51, 95% CI: 0.23-1.10 | DOC+ADT ADT  Any grade Grade ≧3 Any grade Grade ≧3 Any AEs 188 (95.9) 94 (48.0) 163 (79.9) 22 (10.8) Alopecia 110 (56.1) 0 3 (1.5) 0 Fatigue 104 (53.1) 9 (4.6) 58 (28.4) 0 Hot flashes 81 (41.3) 2 (1.0) 118 (57.8) 1 (0.5) Edema 66 (33.7) 2 (1.0) 11 (5.4) 0 Diarrhea 64 (32.7) 3 (1.5) 6 (2.9) 0 Neutropenia 39 (19.9) 31 (15.8) 0 0 FN 14 (7.1) 13 (6.6) 0 0 | | |  |  |
| NCT00764166 Oudard et al. | 2019 | DOC+ADT ADT median PFS 20.3months (19.0-21.6) 19.3 (18.2-20.8) median MFS 106.8months (87.6-122.4) 108.0 (87.6-124.8) median OS NR NR  PFS HR: 0.85, 95% CI: 0.62-1.16, p = 0.31 MFS HR: 1.03, 95% CI: 0.74-1.43, p = 0.88 OS HR: 0.86, 95% CI: 0.56-1.31, p = 0.49 | ADT alone ADT + DOC  Grade 1-2 Grade 3-4 Grade1-2 Grade 3-4 Neutropenia NA 0 NA 60 (48) FN NA 0 NA 10 (8) Asthenia 5 (4) 1 (1) 5(4) 92 (74) Hair loss 0 0 56 (45) 5 (4) Diarrhea 0 NA 42 (34) NA Nail disorder 0 0 49 (39) 1 (1) | | |  |  |
| TOAD Duchesne et al. | 2016 | OS adjusted HR: 0.59, 95% CI: 0.26-1.30, p = 0.19 | Delayed ADT Immediate ADT   Any grade Any grade Cardiovascular 9 (6) 13 (9) CNS 2 (1) 3 (2) | | |  |  |
| NCT00928434 Crawford et al. | 2015 | Time to PSA >4.0 ng/ml Intermittent vs. Continuous ADT: p = 0.5 | Intermittent ADT Continuous ADT   Any grade Any grade Any AEs 159 (91) 205 (90) Hot flushes 89 (51) 137 (60) Fatigue 32 (18) 42 (18) | | |  |  |
| NCIC Crook et al. | 2012 | OS HR: 1.03, 95% CI: 0.87-1.21 CSS HR: 1.23, 95% CI: 0.94-1.60 Time to CRPC HR: 0.81, 95% CI: 0.68-0.98) | Intermittent ADT Continuous ADT  Any grade Grade ≧4 Any grade Grade ≧4 Hot flushes 620 (90) 1 (<1) 641 (93) 0  Impotence 593 (53) 0 608 (53) 0 Lipido 546 (55) 0 541 (51) 0 Fatigue 394 (37) 2 (<1) 385 (43) 3 (1) | | |  |  |
| Others | | | | | |  |  |
| ARTS Schröder et al. | 2013 | Dutasteride Placebo PSA progression rate 13% 17% p = 0.15 Progression 17% 34% p < 0.001  RR: 0.41, 95% CI: 0.25-0.67 | Placebo Dutasteride Any Aes 95 (65) 97 (66) Impotence 6 (4) 0 Altered lipido 4 (3) 1 (1) Breast disorder 4 (3) 10 (7) | | |  |  |
| PROTECT Beer et al. | 2011 | Sipuleucel-T Placebo median PFS 18.0 months 15.4 months  PFS HR: 0.94, 95% CI: 0.64-1.38, p = 0.7 MFS HR: 0.73, 95% CI: 0.34-1.58, p = 0.4 | Sipuleucel-T Placebo  Any grade Grade ≧3 Any grade Grade ≧3 Any AEs 111 (96) 31 (27) 56 (95) 18 (31) Fatigue 52 (45) 0 18 (31) 0 Chills 51 (44) 2 (2) 6 (10) 0 Pyrexia 42 (36) 2 (2) 1 (2) 0 | | |  |  |
| Goluboff et al. | 2001 | Exisulind Placebo 1 yr PFS 22% 67% | Exisulind Placebo  Grade ≧3 Grade ≧3 Abdominal pain 3 NA Dyspepsia 4 NA | | |  |  |

**Supplementary Figure 1.** Risk of bias assessment of the included RCTs


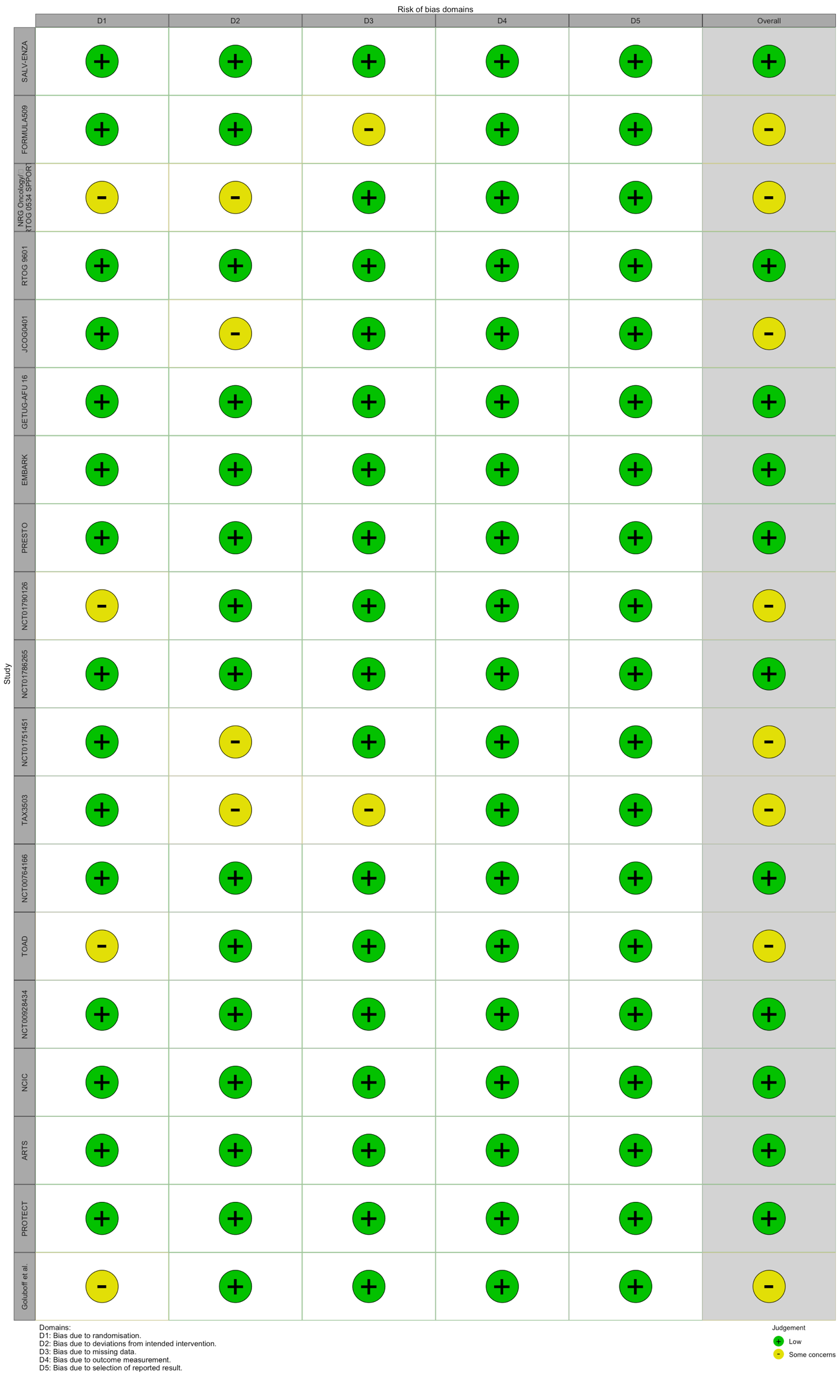


**Supplementary Figure 2.** Funnel plot for publication bias assessment in each analysis: (A) MFS for HT + RT vs. RT, (B) OS for HT + RT vs. RT, (C) OS for DOC + ADT vs. ADT

(A)


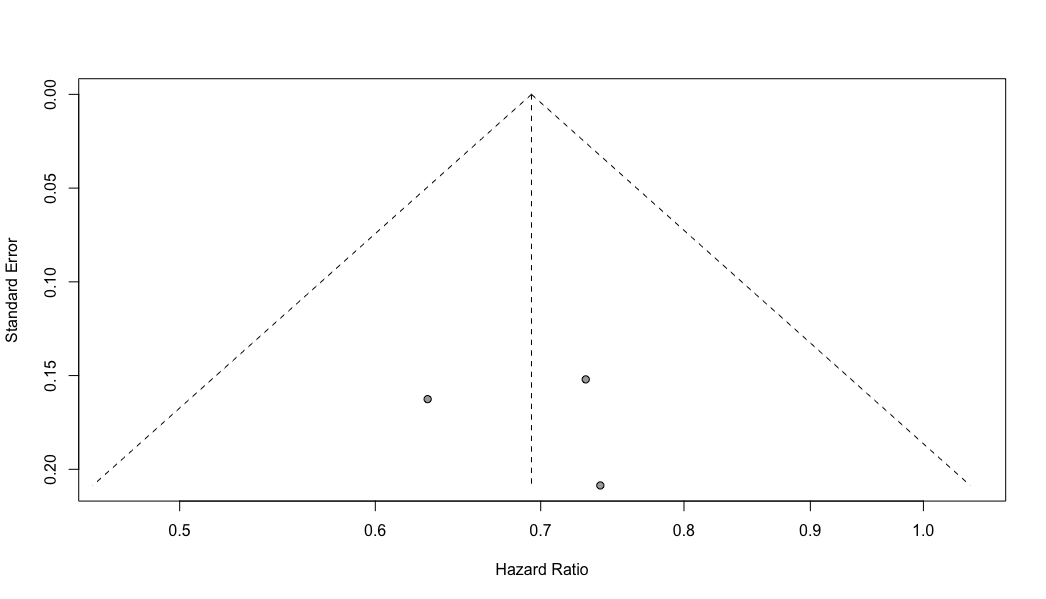


(B)


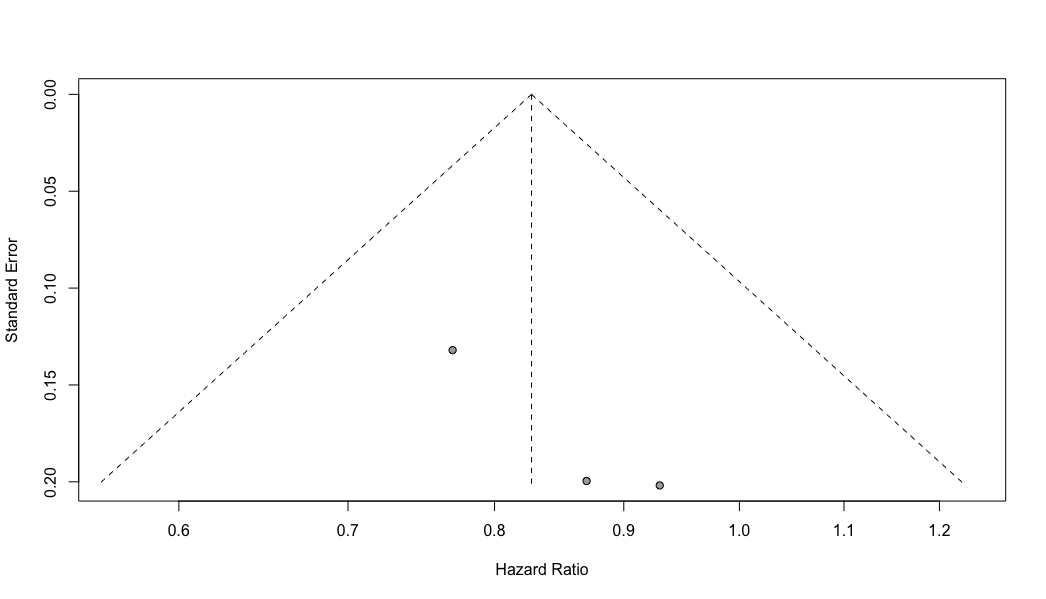


(C)


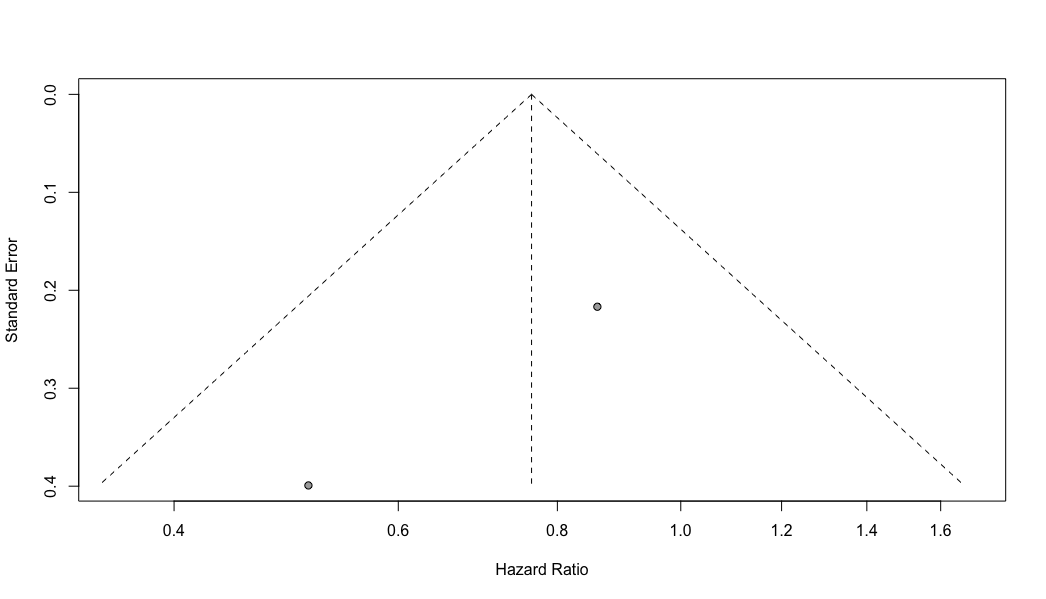


**Supplementary Figure 3.** Network plots of NMA: (A) MFS of radiation-based treatment, (B) MFS of chemotherapy-based treatment, (C) OS of radiation-based treatment, (D) OS of chemotherapy-based treatment

(A)


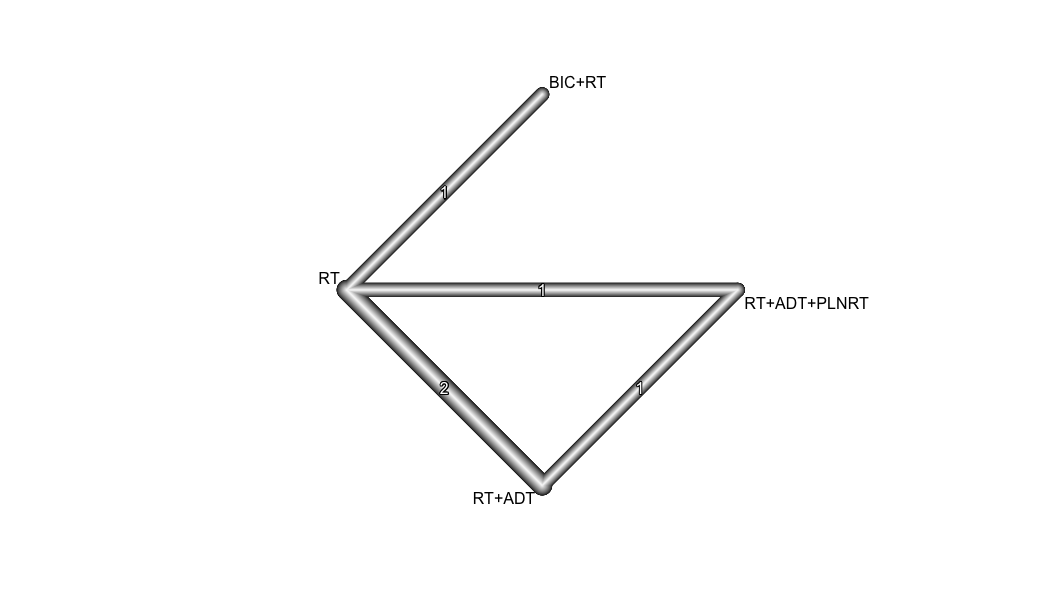


(B)


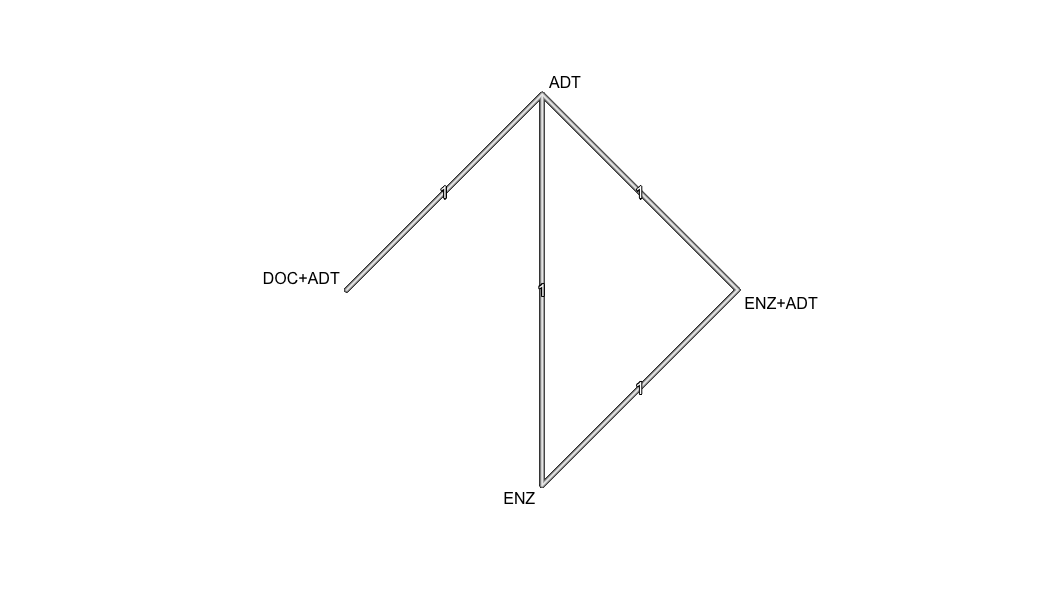


（C）


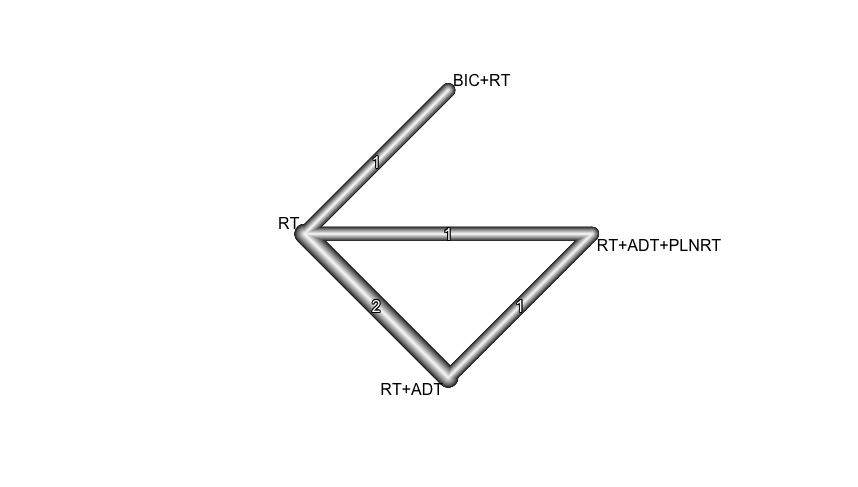


(D)


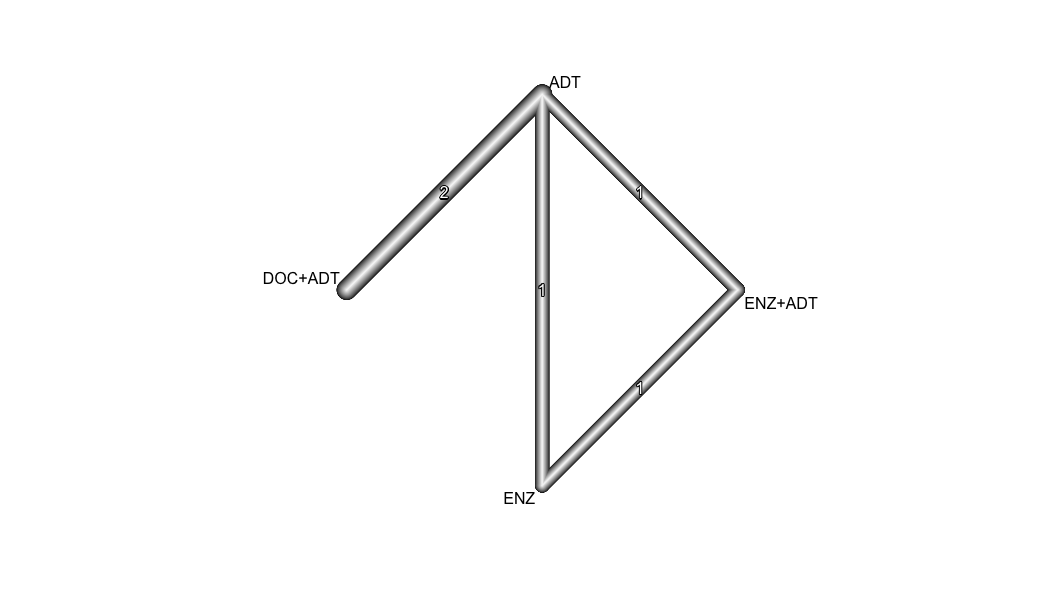


**Supplementary Figure 4.** NMA results for radiation-based treatment on (A) MFS and (B) OS in patients with BCR: Forest plots with RT + ADT as comparator

1. ****MFS
2. **
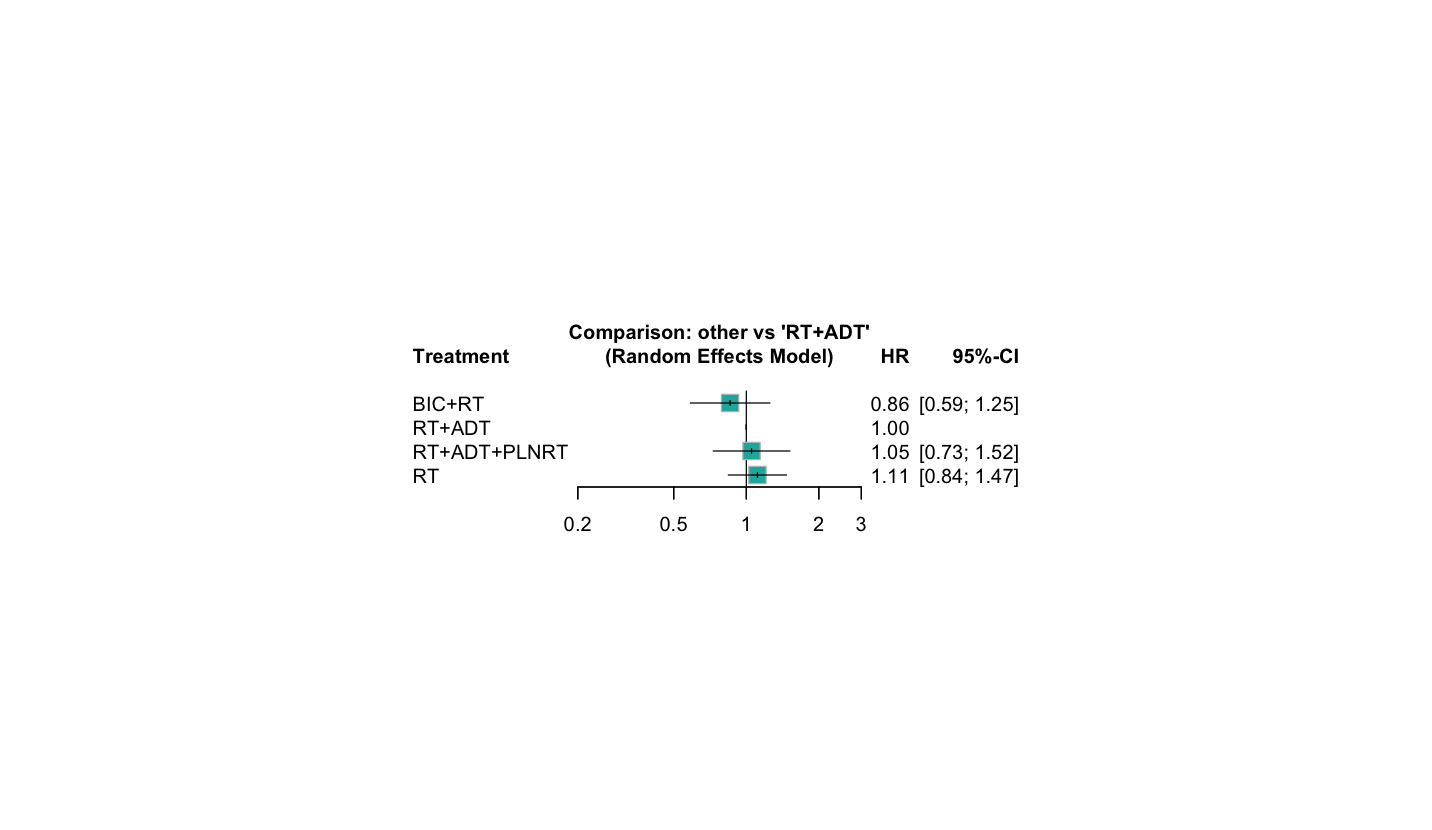
**OS

**Supplementary Appendix 1**. Search strategy for systematic review and meta-analysis

Electronic searches were performed in the following databases to identify eligible studies

1. PubMed (n = 468)
2. SCOPUS (n = 1,305)
3. Web of science (n = 879)

There were no language or publication period limitations.

PubMed

1. Search: prostate cancer [MeSH Terms] Sort by: Most Recent
2. Search: prostate cancer [Title/Abstract] Sort by: Most Recent
3. Search: biochemical recurrence [MeSH Terms] Sort by: Most Recent
4. Search: biochemical recurrence [Title/Abstract] Sort by: Most Recent
5. Search: randomized controlled trial [Publication Type] Sort by: Most Recent
6. Search: RCT [Title/Abstract] Sort by: Most Recent
7. Search: prospective studies [Publication Type] Sort by: Most Recent
8. Search: prospective studies [Title/Abstract] Sort by: Most Recent
9. Search: ((#1) OR (#2)) AND ((#3) 0R (#4)) AND ((#5) OR (#6) OR (#7) OR (#8)) Sort by: Most Recent

SCOPUS

TITLE-ABS-KEY (“prostate cancer”) AND TITLE-ABS-KEY (“biochemical recurrence”) AND TITLE-ABS-KEY (“randomized controlled trial”) OR TITLE-ABS-KEY (“RCT”) OR TITLE-ABS-KEY (“prospective study”)

Web of science

1. TS = (prostate cancer)
2. TS = (biochemical recurrence)
3. TS = (randomized controlled trial)
4. TS = (RCT)
5. TS = (prospective study)
6. (#1) AND (#2) AND ((#3) OR (#4) OR (#5)

**Supplementary Appendix 2**. AMSTAR-2 checklist


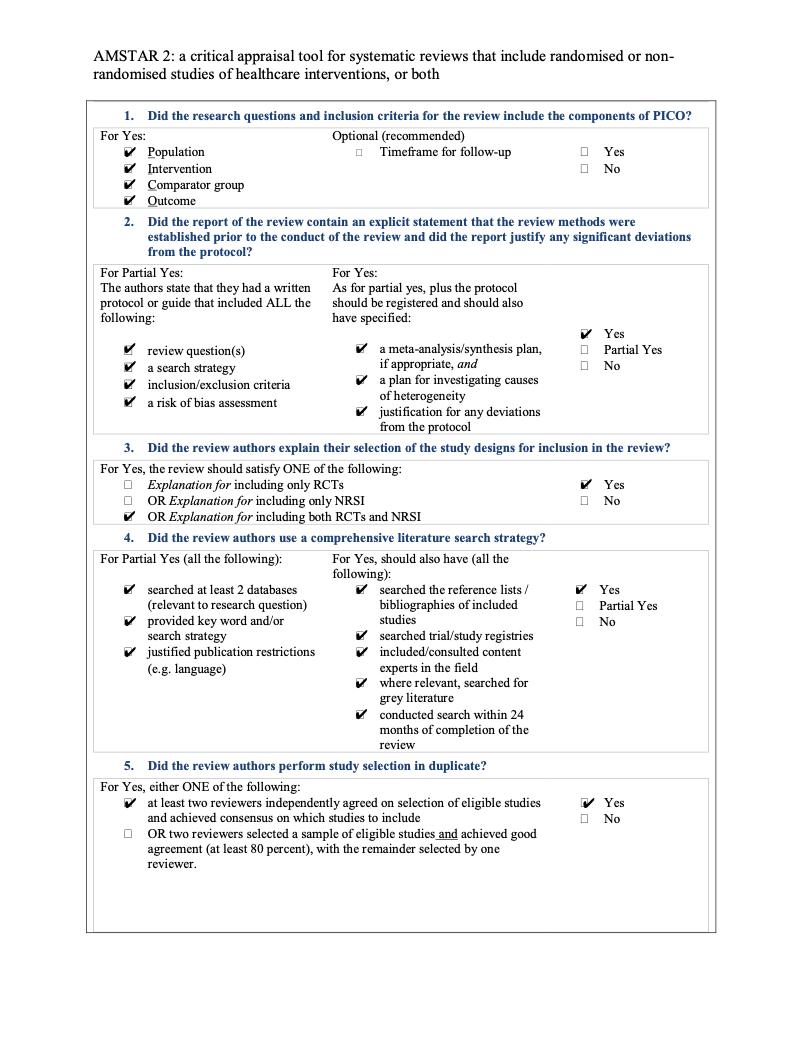


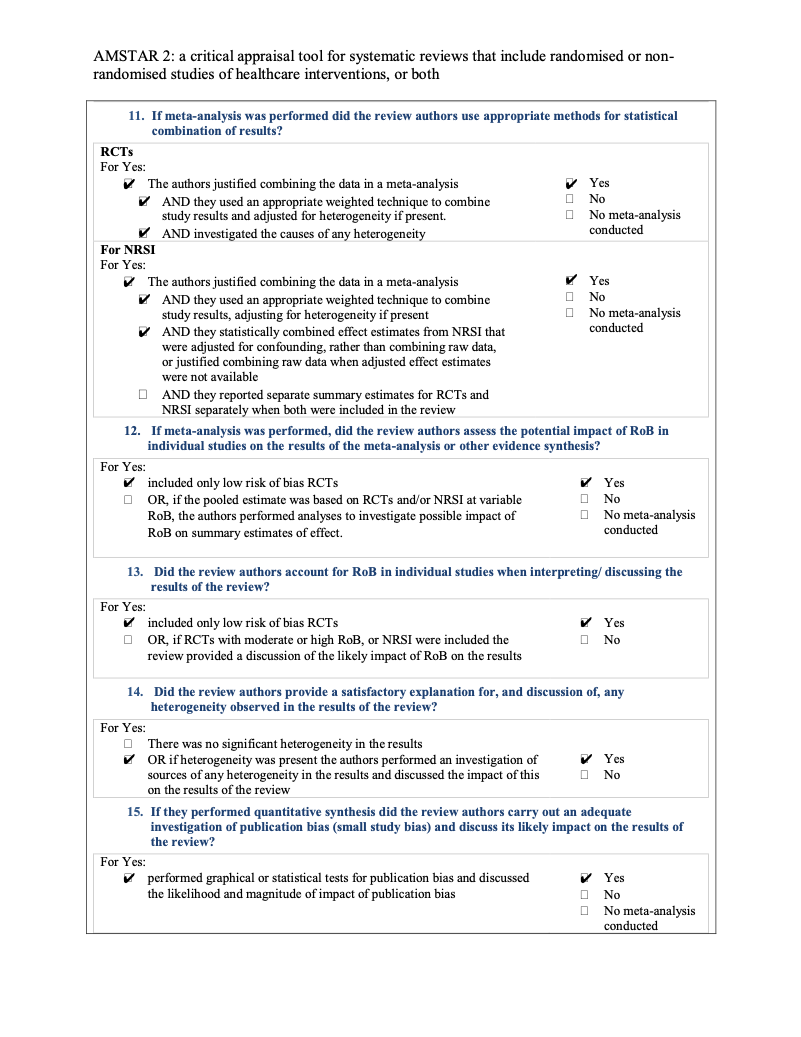


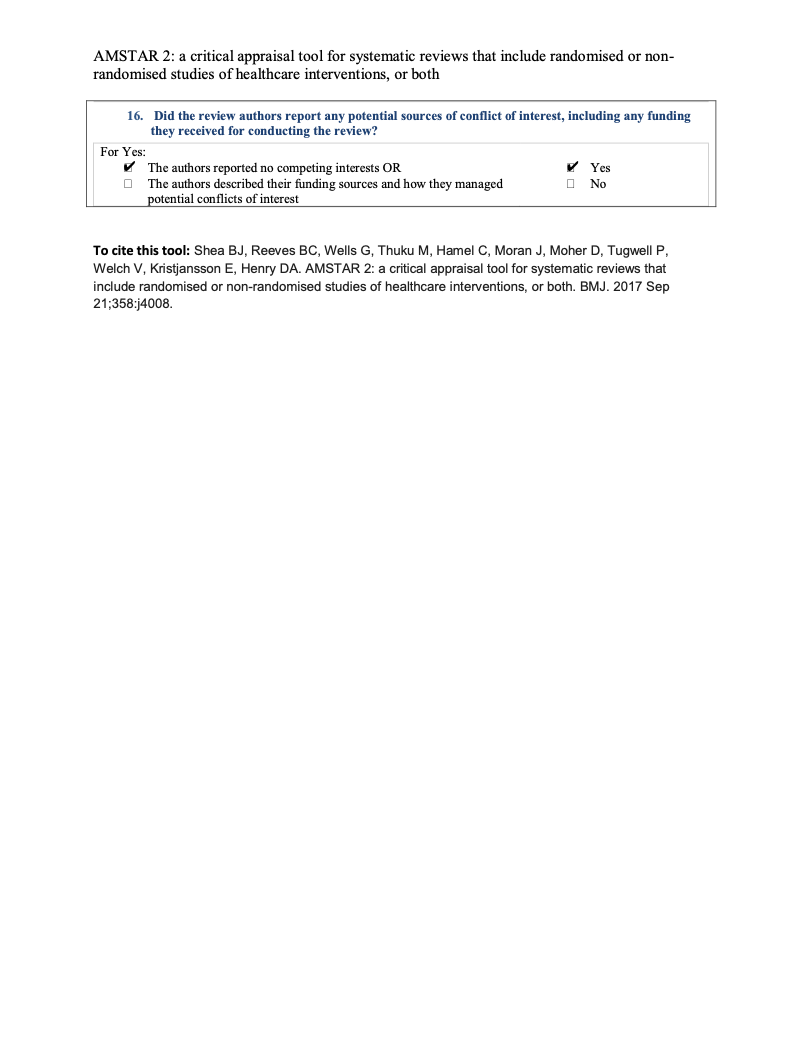

Supplement: Supplementary file 1 — Supplemental Material [file 41391_2024_890_MOESM1_ESM.docx]
